# Journeying to the past: time travel and mental time travel, how far apart?

**DOI:** 10.3389/fpsyg.2023.1260458

**Published:** 2023-12-28

**Authors:** Marina Trakas

**Affiliations:** National Scientific and Technical Research Council (CONICET), Buenos Aires, Argentina

**Keywords:** time travel, mental time travel, personal memory, episodic memory, past

## Abstract

Spatial models dominated memory research throughout much of the twentieth century, but in recent decades, the concept of memory as a form of mental time travel (MTT) to the past has gained prominence. Initially introduced as a metaphor, the MTT perspective shifted the focus from internal memory processes to the subjective conscious experience of remembering. Despite its significant impact on empirical and theoretical memory research, there has been limited discussion regarding the meaning and adequacy of the MTT metaphor in accounting for memory. While in previous work I have addressed the general limitations of the MTT metaphor in explaining memory, the objective of this article is more focused and modest: to gain a better understanding of what constitutes MTT to the past. To achieve this objective, a detailed analysis of the characteristics of MTT to the past is presented through a comparison with time travel (TT) to the past. Although acknowledging that TT does not refer to an existing physical phenomenon, it is an older concept extensively discussed in the philosophical literature and provides commonly accepted grounds, particularly within orthodox theories of time, that can offer insights into the nature of MTT. Six specific characteristics serve as points of comparison: (1) a destination distinct from the present, (2) the distinction between subjective time and objective time, (3) the subjective experience of the time traveler, (4) their differentiation from the past self, (5) the existence of the past, and (6) its unchangeability. Through this research, a detailed exploration of the phenomenal and metaphysical aspects of MTT to the past is undertaken, shedding light on the distinct features that mental time travel to the past acquires when it occurs within the realm of the mind rather than as a physical phenomenon. By examining these characteristics, a deeper understanding of the nature of mental time travel is achieved, offering insights into how it operates in relation to memory and the past.

## 1 Introduction

Over time, various memory models, influenced by different analogies and metaphors, have guided empirical research in the field of memory. Spatial models were prominent in memory research for much of the twentieth century (Roediger, 1980; Draaisma, 2000), until around 1980 when a new metaphor emerged: the mental time travel metaphor (MTT) (Tulving, 1983, 1985; Suddendorf and Corballis, 1997, 2007; Gardiner, 2001; Tulving, 2002a,b; Tulving, 2005). On the one hand, the conceptualization of memory as a MTT capacity shifted the focus from the spatial nature of memory to its temporal dimension (see Trakas, 2022 for more details). It conceptualized future thinking and planning as a movement from the present to the future, and episodic memory or memory of past experiences as a movement from the present to the past, both involving a traversal through *subjective time*. Subjective time pertains to the temporal dimension within our consciousness that is self-referential, encompassing reflections on past and future events involving the subject as an observer or participant (Nyberg et al., 2010). In what appears to be the first reference to MTT, the psychologist and neuroscientist Endel Tulving (1985) wrote: “A normal healthy person (…) is capable of becoming aware of her *own* past as well as her *own* future; she is capable of mental time travel, roaming at will over what has happened as readily as over what might happen, independently of physical laws that govern the universe” (Tulving, 1985, p. 5).^1^ On the other hand, the conceptualization of memory as a MTT capacity shifted the focus from the internal processes of memory, involving the encoding and retrieval of information and the quantification of the information retrieved, to our subjective experience of memory (Koriat and Goldsmith, 1996; Wheeler et al., 1997). While the internal processes of memory were traditionally approached from a third-person perspective, the conceptualization of memory as MTT aimed to reframe our understanding of memory by emphasizing a first-person approach centered on the subjective conscious experience of remembering (Gardiner, 2001). This novel metaphor, centered on temporality and consciousness, spurred the development of new research paradigms and protocols to study human memory, particularly in the early twenty-first century. It also played an important role in the philosophy of memory, giving rise to the simulation theory of memory (Michaelian, 2016), which is presented as an alternative to causal theories of memory that rely on causal mechanisms and memory traces.

Despite the significant impact of the MTT metaphor on empirical and theoretical research on memory, there has been little discussion about its meaning and its adequacy to account for memory. While in previous research, my focus has been on assessing its overall adequacy by addressing the general limitations of the MTT metaphor in explaining memory (Trakas, 2022), this article seeks a narrower and more modest objective. Given the limited analysis of the nature and experience of MTT itself, the goal is to gain a better understanding of what constitutes a time travel to the past *within the mind*. To accomplish this task, I consider the concept of time travel (TT), and draw a parallel between the two. MTT is fundamentally derived from the concept of TT, which not only predates the idea of MTT and is more familiar to us but has also been the subject of extensive scholarly discourse. MTT, in essence, posits a linkage between TT and memory, suggesting that memory can be regarded as a form of TT that occurs *within the mind*. Therefore, delving into the attributes of TT to the past to gain insights into the nature of MTT to the past and examining whether certain memories, as discussed in the literature, exhibit such characteristics, appears to be a promising initial step in advancing our comprehension of the application of the MTT metaphor to memory.

The analysis is exclusively focused on TT and MTT to the *past* for three reasons. Firstly, it is part of a larger project aimed at comprehending and evaluating the MTT metaphor and other memory metaphors that have influenced the scientific research on memory. Given the importance that this metaphor has acquired in the last years, particularly regarding theories that blur or do not clearly differentiate between imagination and memory (Michaelian, 2016; Addis, 2018, 2020), it becomes imperative to carefully examine what it really means to conceive memory as a form of mental time travel to the past. Secondly, the metaphor of MTT was originally introduced in the field of memory research for the purpose of understanding memory (Tulving, 1983, 1985; for more details, see Trakas, 2022). Restricting the scope of our analysis to past-focused MTT enables us to grasp—and potentially assess—the metaphor within the context of its primary intention: explaining the workings of memory. Lastly, it is not inherently clear that MTT to the past would possess analogous characteristics to MTT to the future (see, for example, Debus, 2014; Barkasi and Rosen, 2020; De Brigard, 2023, for a critique of mental time travel as a brain network system). While investigating this topic would be intriguing, it would constitute a separate project, one that could nevertheless significantly build upon the initial step taken here.

Hence, my aim in this paper is to offer a detailed analysis of the unique characteristics of MTT to the past by comparing and contrasting it with TT to the past. Exploring both the similarities and differences between the two will be a central focus of this paper, allowing us to better understand the phenomenal as well as the metaphysical aspects of MTT to the past. It is important to note that this research is not purely speculative. While TT is not currently an existing phenomenon, there is a generally accepted consensus regarding its fundamental characteristics, as I elaborate upon in the following sections. These foundational characterizations can guide our comprehension of what to expect from a TT to the past that is mental. Additionally, I draw upon empirical evidence from the memory literature to inform the characterization of MTT to the past. While certain speculative and philosophical aspects are involved in this characterization, the majority of it is rooted in empirical findings.

## 2 General overview on time travel

I will not delve here into the history and initial developments of the concept of MTT (see Trakas, 2022). However, it is important for the subsequent discussion to provide a brief and general overview of the idea of time travel (TT). The concept of TT emerged in the late nineteenth century and gained popularity through Wells’ science fiction novel *Time Machine* (Wells, 1895). As Bigelow explains:

“the exponential explosion of time travel stories in the popular media, beginning late in the nineteenth century, is an indication that a very new conception of time is brewing in the Zeitgeist. The utter absence of any time travel stories whatsoever prior to the nineteenth century is a profoundly puzzling fact … I suggest that this is at least partly explained by the utter universality of presentism prior to the nineteenth century and by the utter absence of any rivals to presentism.” (Bigelow, 1996, pp. 35–36)

Presentism, the theory that only the present is real, implies that time travel (TT) is not possible in principle. The prevalence of presentism prior to the nineteenth century could account for the absence of the concept of TT itself. However, with the emergence of the theory of relativity, alternative conceptions of time began to be seriously considered in physics and philosophy. It was only in the late 1970s that the concept of TT gained significant attention in the philosophical literature, particularly following the influential paper by Lewis titled *The paradoxes of time travel* (1976). As Bernstein (2022) explains, Lewis initially aimed to capture TT as a genuine form of travel akin to what was depicted in the science fiction of his time.

Although TT is an idea that has been present in science fiction for over a century and in philosophy for almost 50 years, it can still pose a challenge when used as a starting point to better understand MTT. TT does not refer to an existing physical phenomenon. While many philosophers and physicists argue for the logical and physical possibility of TT, and there is a considerable consensus on how natural laws might allow for its occurrence (Rawls and Miller, 2000; Dainton, 2001; Bardon, 2013; Miller, 2017a; Hawking, 2018), it remains an idea with varying conceptualizations in philosophy, and particularly so in science fiction. This could be seen as an argument against using TT as a means to better understand MTT. One could argue that since TT does not refer to an empirically studyable physical phenomenon, its characteristics cannot shed light on the conceptualization of MTT. The multiple hypothetical characterizations of TT would result in equally varied characterizations of MTT, rendering the comparison futile. While this argument acknowledges the limitations of the comparison, it does not render the comparison completely fruitless. There are commonly accepted general characterizations of TT in the philosophical literature, particularly in orthodox theories of time, that can provide insights into what MTT might be. Given that TT has been extensively discussed and is an older idea than MTT—and one that we are more familiar with, it undoubtedly offers perspectives on the possible characteristics of a mental form of TT. From this point onward, the focus will be solely on these general characteristics of TT, without delving into debates on its specificities found in the philosophical literature (many of which revolve around puzzles and paradoxes related to specific TT scenarios). I consider the following as key characteristics of TT to the past:

- The destination of TT is a different time period from the present;
- There is a distinction between the subjective time experienced by the time traveler and the external or objective time;
- The time traveler can experience the past for the first time and/or re-experience it;
- The time traveler is distinct from their past self;
- The past is considered to exist;
- The past is ultimately unchangeable.

In the subsequent sections, I delve into each of these characteristics in greater detail to establish the resemblances and disparities with MTT to the past, always with the ultimate goal of providing a comprehensive and detailed characterization of MTT.

## 3 Traveling to a temporal destination different from the present

Typically, TT is understood as the removal of the traveler from the usual flow of time, resulting in their appearance earlier or later in the timestream (Keller and Nelson, 2001; Dyke, 2005; Hales, 2010). In essence, TT takes a familiar spatial concept, travel, and applies it to the temporal realm. Analogous to traveling through space, TT involves a disparity between the temporal coordinates of the starting point and of the destination, that is, the place to which the person travels or is sent. Generally, TT entails moving from a specific time point, t0, to either a time earlier, t−1, or later, t + 1.

Another aspect of the destination of TT pertains to the knowledge possessed by the time traveler in relation to their journey. This is not a topic of theoretical discussion, and we need to rely on science fiction to explore the different possible scenarios. In many fictional situations, the traveler is aware of the specific temporal coordinates of their destination and may even consciously and intentionally choose them, such as it happens in many films like *Back to the future*, where characters use the DeLorean time machine created by Doc Brown. However, this does not always need to be the case. Time travel can be involuntary, with the traveler gaining knowledge of their temporal destination either upon arrival or after spending some time there. This happens to Mikkel from the German series *Dark*, who unintentionally travels to the past through a cave wormhole and only becomes aware of his temporal displacement when he discovers the date through a newspaper. It is even possible for time travelers to be unable to obtain the exact temporal coordinates due to variations in how time is measured or the absence of temporal measurement altogether (such as going back to the Mesozoic Era). Therefore, while knowing the spatio-temporal coordinates of the destination can be common, at least when considering science fiction time travel scenarios, it does not need to be essential to TT. Similarly, while involuntary TT is often depicted in science fiction, the likelihood increases that, once TT mechanisms become widely known, individuals would intentionally engage in TT, leading to voluntary TT being more common than involuntary. Additionally, being aware that one is traveling through time or realizing it upon reaching the destination may not be crucial either. Nevertheless, in all instances, regardless of whether it is known or unknown to the traveler, intentional or unintentional, the destination remains highly precise, corresponding to specific spatio-temporal coordinates.

Considering that a distinction between the origin and the destination, especially in terms of temporal coordinates, is fundamental to the concept of TT, a mental travel occurring in subjective time should also possess this essential characteristic. We should experience, if not going, at least arriving at a different time than the present. This is akin to what often occurs when we recollect significant events from our lives, for example, when I reminisce about the swim I had last summer with my friends in front of Trakai Castle. While seated on a couch and allowing my mind to wander, my attention shifts away from current tasks, task-related thoughts, and stimuli in my immediate surroundings, to that day when I swam in front of Trakai Castle, *as if* I was traveling back there, living again the adventure I had experienced. This attentional shift, which is characteristic of mind-wandering, is known as perceptual decoupling (Schooler et al., 2011; Smallwood and Schooler, 2015): the attention to external stimuli is reduced and greater attentional resources are allocated toward self-generated thoughts, such as memories.

Several factors contribute to the vividness and sense of reliving the past during this form of mind-wandering focused on past experiences. Visual imagery, emotional processes, and self-referential thinking all play a role (Palombo et al., 2018; see section 5 for more details). However, in many instances, perceptual decoupling occurs after perceiving a cue in the environment that triggers the memory or prompts a search for the memory (Conway and Pleydell-Pearce, 2000). For example, revisiting Lithuania and witnessing the castle again can detach me from my present environment and mentally transport me back to the day when I was swimming in front of that castle with my friends. Sometimes, material objects and contexts not only serve as triggers but also become integral parts of the MTT experience (see sections 5 and 7). This also applies to conversations, as in joint remembering, where individuals mentally travel back in time by collectively recalling a shared past event (Harris et al., 2011). Likewise, recounting a personal experience to someone else can lead to a sort of *embodied* MTT experience, where the storyteller reenacts past movements or performs embodied symbols and metaphors that refer to the past (Trakas, 2021a: see section 5). Consequently, mind-wandering, material objects, environments, and even conversations have the power to detach us from our present tasks and concerns, allowing us to *travel* mentally to specific points in our personal past. In this regard, the difference between origin and destination characteristic of TT, and by extension of MTT, appropriately captures the conscious experience and phenomenal aspects of *certain* memories of personal past experiences. I am not asserting that all memories of personal experiences necessarily exhibit this characteristic, implying a sense of being removed from the present and traveling back to the past where the experience occurred. As argued in Trakas (2022), *many* memories of personal past events deviate from presenting this essential trait of MTT. Nonetheless, the aim of this article is to explore the nature of MTT, so the following analysis is restricted to memories that, at a minimum, adhere to this condition—an aspect fundamentally inherent to the concepts of TT and, consequently, MTT.

There are also correlations between aspects of our memories and the different knowledge that the time traveler can possess regarding their TT journey. The differentiation between intentional TT to a known location and unintentional TT mirrors the distinction between voluntary and involuntary memories, which arise from distinct retrieval mechanisms. Voluntary memories are consciously recalled in a strategic and goal-directed manner, and are experienced as such, while involuntary memories come to mind spontaneously, with no preceding conscious attempt at retrieval, resulting in a subjective experience of unintended recollection (Berntsen, 2009). “Involuntary mental time travel” is then the “mental time travel that takes place spontaneously—that is, with no preceding conscious attempt at mentally projecting oneself forward or backward in time” (Berntsen and Jacobsen, 2008, p. 1093). While cognitive psychologists previously regarded voluntary memories as the norm and involuntary memories as exceptional, empirical evidence now contradicts this notion. Involuntary memories and MTTs to the past are highly common in daily life, with their frequency being comparable at least to that of voluntary memories (Rubin and Berntsen, 2009; Berntsen, 2021). Considering that intentional TT is likely the more prevalent form of time travel, as argued earlier, while unintentional TT is probably less frequent, this characteristic of MTT could point to a potential—but not radical—distinction between TT and the MTT enabled by memory.

On the other hand, certain involuntary memories associated with post-traumatic stress disorder (PTSD) could exemplify the instances where the time traveler neither chooses to engage in time travel nor initially realizes (for a short period of time) that they have actually traveled through time. In some cases of PTSD flashbacks, there is a distortion in the perception of time, causing the recollection of past traumatic events through visual imagery to be processed as if they are happening once again in the present moment. These intrusive and uncontrollable traumatic memories often exhibit a feeling of “nowness,” particularly before undergoing treatment (Ehlers et al., 2004; Hackmann et al., 2004; Brewin, 2015). This *hallucinatory* nature (Ribot, 1907) can profoundly impact behavior, with individuals displaying signs of terror, experiencing autonomic symptoms like sweating, and even engaging in bodily movements (Brewin and Holmes, 2003; Holmes and Mathews, 2010). For instance, a woman who had been previously attacked by a bull had a flashback at a petrol station and, without being aware that she was remembering, unintentionally sprayed another customer with diesel fuel (Ehlers et al., 2004). These types of memories serve as a prime example of MTT that occurs without being consciously experienced as such, at least during the brief duration of the flashback (also refer to sections 5 and 6).

Finally, another similarity between TT and many of our memories lies in the fact that knowing the precise spatio-temporal coordinates of the destination is not essential for either TT or MTT. Although Tulving (1983) initially defined episodic memory as “information about temporally dated episodes or events and the temporal–spatial relations among them” (p. 143), temporal-contextual information is not considered to be crucial for remembering the personal past. This is because we often struggle to accurately and precisely locate past events in time, and our temporal judgments are often prone to inaccuracies (McCormack, 2001). Hence, for us to mentally experience traveling back in time during recollection, it suffices, at least in terms of temporal information, that the event represented as our destination feels as if it occurred at a time earlier than the present. I will delve into the concept of the “feeling of pastness” in section 5.

It appears, therefore, that MTT exhibits some similarities with TT concerning this characteristic. Nevertheless, important differences also exist, which are more significant than the potential frequency of voluntary and involuntary instances of MTT. More precisely, the destinations of many of our MTTs differ radically from those of traditional TTs.

The primary distinction that can naturally arise is that while theoretically we can travel back to any point in time, even before our birth, the general limits of MTT are bound by our personal history. We cannot mentally journey to a time prior to our own existence. We can certainly imagine scenes from the First World War, for example, drawing upon knowledge obtained through testimonies and historical accounts. We can even imagine ourselves participating in those scenes we have constructed. However, when we embark on this imaginative endeavor, we do not actually transport ourselves mentally to those moments and vividly re-experience the fabricated events, because we do not experience them as being part of our subjective time (further elaboration on this topic will be presented in section 5). MTT is bound to personal, subjective time, and this by definition: “mental time travel refers to conscious experience of remembering the *personal* past and imagining the *personal* future” (Nyberg et al., 2010, p. 22536). This represents a crucial difference between the potential destinations of TT and MTT. Nevertheless, this distinction requires some nuance.

Certain individuals claim to possess memories of past lives, possibly driven by magical ideation, spiritual motivations (McNally, 2012), and/or source-monitoring errors (Peters et al., 2007). Limited research has been conducted on these types of memories. It is conceivable that the feeling of familiarity experienced by some individuals leads them to internally accept events generated within their minds as memories (Peters et al., 2007), without necessarily involving the phenomenal experience of mentally traveling back in time and reliving those events. However, if memories of past lives are accompanied by a strong sensation of traveling back in time, it could be argued that people, or at least some individuals, can mentally revisit time coordinates preceding their own birth. Nevertheless, these cases would remain exceptional instances of MTT, as memories of past lives are exceptional occurrences not only within Western culture but also in cultures where the belief in reincarnation is prevalent (Meyersburg et al., 2014). Furthermore, even in these exceptional cases, the limits imposed by their *personal* history would still restrict the potential destinations they can reach. They cannot travel to a time that is not self-referential, meaning it does not manifest itself or feel like a period where they exist in some form of life. This holds true even if it turns out to be the product of imagination. However, this type of response might imply that TT ultimately faces limitations similar to those faced by MTT. If the past destinations of time travelers already include the actions and existence of the time travelers themselves, as is generally assumed in orthodox theories of time and TT (see section 8 for more details), time travelers would also be unable to journey beyond their “personal history.” These past actions and existences are already part of their history, even if they are unaware of it or have yet to experience them. Even if individuals travel five centuries before their birth, their past actions and existence are already integral parts of the history of that period; thus, strictly speaking, they cannot travel outside their personal history. Although in the case of TT, the personal history is metaphysical, whereas in MTT, the personal history is phenomenal and refers to the history felt by the person as their own, personal history sets a limit to both TT and MTT. It can be then suggested that the scope of destinations in both TT and MTT may not ultimately differ as much as initially perceived.

Although this initial distinction can be questioned and may not be as radical as it seems, there are two other notable differences between the destinations of TT and MTT that hold greater significance. Firstly, while the destinations of TT are precise spatio-temporal coordinates, such as 3:15 p.m. on July 15, 1990, most of our memories do not possess this characteristic. The “destinations” or intentional objects of MTT are not literal recordings of past experiences, akin to an excerpt from a video tape. Even experience-near events are mentally replayed at a faster rate compared to the actual event duration (Jeunehomme and D’Argembeau, 2019), and are always constructed from fragments of prior experiences already integrated with generic knowledge (Conway and Pleydell-Pearce, 2000; Conway, 2009: more in section 8). Furthermore, many times these destinations do not refer to experience-near events; they involve abstracted, summarized, and generalized events that span extended periods of time. “Memory destinations” can occur at multiple timescales and levels of specificity. Sometimes we travel back to complex events that encompass a series of simple events, such as “going to the airport” or “a trip to Malaysia.” Other times, the destination encompasses repeated events that have occurred over extended periods, like “summers at the beach,” or even an entire life-time period, such as “life in the White House in Newtown” or “childhood” (Linton, 1986; Barsalou, 1988; Conway and Pleydell-Pearce, 2000; Trakas, 2019a; D’Argembeau, 2020; Andonovski, 2021).

Moreover, the destinations of our MTTs are not typically stable and punctual; they fluctuate between simple and more complex, abstract events. As Neisser explains, “recalling an experienced event is a matter not of reviving a single record but of moving appropriately among nested levels of structure” (Neisser, 1986, p. 71). The destination is therefore “movable”: in free recall protocols, memories of simple events are often invoked alongside memories of complex and general events (Barsalou, 1988). When we mentally travel back in time, we can swiftly and flexibly navigate different timescales and layers of autobiographical representations. This navigation occurs vertically, transitioning from lifetime periods or general events to zoom in on specific events, and vice versa, as well as horizontally, “jumping” to a point in time and “moving” forward chronologically to explore events or periods (D’Argembeau, 2020). Consequently, this distinct nature of the destination marks a significant difference between TT and the type of MTT we engage in when we remember.

Secondly, the destinations of MTT differ so profoundly from the destinations of TT that, in some instances of MTT, the destination can be non-existent, in the sense that it may not have occurred in spacetime and may ultimately be a product of imagination. This is evident in cases of memories of previous lives or in other instances of complex and full false memories of entire events. Even when the remembered event did not occur, our phenomenal experience can still exhibit the characteristics of MTT. We can place that event in temporal coordinates distinct from the present, as we mentally move away from the present toward this imaginary yet convincingly real past destination. Unlike TT, where the past destination must exist in spacetime for us to travel there, MTT allows us to explore and engage with a past that may only exist within our consciousness (see section 7).

## 4 Difference between the personal time of the time traveler and the external time

Another important characteristic of TT, which has been highlighted since Lewis (1976) as a defining feature (though also subject to criticism: see Bernstein, 2022), is the divergence between personal time and external time. In ordinary life, our personal time aligns with the external time, but in TT, the personal time of the traveler differs from the external time. Various interpretations of this characteristic have been proposed. One interpretation emphasizes the time gap between departure and arrival. In TT, the objective time measured in the surrounding world between t0 and t−1 (e.g., 10 years) differs from the duration of the journey experienced by the time traveler from t0 to t−1 (e.g., 10 min) as measured by their wristwatch (Dainton, 2001; Hunter, 2004; Bernstein, 2022). The time of departure and the time of arrival may also be separated by the direction of time: in objective time, t−1 precedes t0 by a difference of 10 years, whereas in the subjective time of the time traveler, t−1 follows t0 after 10 min (Dainton, 2001; Bernstein, 2022, although t−1 may precede t0 if t−1 was experienced by the time traveler, which poses certain problems, as discussed by Grey, 1999). This notion can also be understood in terms of the order of events (Keller and Nelson, 2001; Markosian, 2020): the time traveler experiences the order of events in their life according to standard causal patterns (e.g., disappearing in t0 and later reappearing in t−1), and this order differs from the objective order of events (the reappearance in t−1 precedes their disappearance in t0), as well as the order of events experienced by non-time-travelers (e.g., the time traveler’s disappearance followed by a world without the time traveler).

In MTT, there should also be a distinction between the personal time of the traveler and the external time. This discrepancy is indeed noticeable in many acts of recollection. While we are engrossed in current tasks and thoughts related to those tasks, we do not perceive a temporal mismatch. Although the passage of time may be subjectively slower or faster than its actual pace (Droit-Volet, 2013; Allman et al., 2014), our actions and thoughts remain rooted in the present, along with our experience of them. When we engage in mental time travel, our subjective perception of time shifts from the present to the past, creating a discrepancy with the ongoing flow of objective time—a flow that we may still perceive, albeit in the background of our temporal experience. Tulving (2002a,b) coined the term “chronestesia” to describe the form of consciousness that allows human beings to experience and reflect upon a personal time within which their experiences unfold. As human beings possess an awareness of the temporal dimension of their existence, they can freely move through this *subjective* time and embark on mental travels to both their future and their past.

The distinction between subjective time and objective time in MTT is evident in various interpretations of this characteristic attributed to TT. To illustrate, let us consider my recollection of swimming near Trakai castle with friends. In this instance, the objective time elapsed between that past moment and the present moment, when I once again see Trakai castle (3 months), differs from the subjective time experienced between these two temporal coordinates (1 s), as measured by my wristwatch. Furthermore, the discrepancy extends to the direction of time: in objective time, the act of swimming near Trakai castle precedes my present perception of Trakai by 3 months, whereas in the subjective time of the rememberer, this past event follows my present perception after a mere 1 s. This disparity also accounts for variations in the sequence of events between objective time and subjective time.

The distinction between the subjective, personal time of the time traveler and the external time remains a consistent characteristic in MTT. However, there may be subtle distinctions to consider. As discussed in the previous section, the nature of subjective time itself grants a level of flexibility to the mental time traveler that may not be available to the time traveler. MTTs can occur rapidly, at varying timescales, and often entail no discernible cost, in the sense of “cognitive cost,” for the traveler. Furthermore, MTT exhibits scale-invariance (Maylor et al., 2001), meaning the rate of event production, or the speed of access to the past event, remains constant across different timescales, whether recalling recent activities, events from the past week, or even events from years ago. If TT requires different amounts of time depending on the temporal destination, this would signify another distinction from MTT.

## 5 Experiencing and re-experiencing the past

While there is a considerable amount of philosophical literature on TT that delves into specific scenarios like the grandfather paradox, there has been limited exploration of the conscious experience itself of TT for the time traveler. However, employing conceptual analysis, we can speculate on the different forms of conscious experiences based on the potential destinations in a block-universe.

In cases where the destination is a point in time before the traveler’s birth or a time when they were alive but lacked first-hand experience, time travelers encounter entirely new experiences never before lived. Although they may possess prior knowledge of those past events, including their own involvement, acquired through testimony and historical accounts, it is only through traveling to the past that they can have a first-hand experience of those events. Because the first-hand experience of these events occurs only after traveling back in time, these events cannot be remembered. Except in cases where time travelers are unaware of their time travel or journey to a period about which they lack semantic knowledge, they know that they are in the past and could, in principle, experience those events as past in a certain minimal sense, although not as part of their *personal* past. Moreover, a feeling of temporal and spatial *presence* of the past, that is what is finally perceived, would encompass their entire experience: they are immersed in a reality of objects that hold ergonomic significance for them and are perceived as existing independently of their mind (see Nanay, 2016; Rosen and Barkasi, 2021), although belonging to the past—and experienced as such.^2^ Similar to the aforementioned cases, instances where time travelers travel to a time when they were alive and had first hand experience, yet do not remember it, also result in a new experience for the traveler. However, it should be noted that memories can resurface once they begin to reexperience their forgotten past. If the past is suddenly recalled, the experience becomes akin to cases where the destination is a time when they were alive, had first-hand experience, and remember it. In such instances, the traveler does not have a new experience but rather relives and reexperiences their personal past as it originally occurred, albeit from a third-person perspective (at least visually: see section 6) rather than a first-person perspective, as it was experienced by their former self. The traveler literally observes their past self and past experiences as an external observer. In this context, the sense of presence of the events that surrounds the time traveler becomes deeply intertwined with a strong sense of what is known in the philosophical literature as a feeling of pastness (Russell, 1921; Broad, 1925; Woozley, 1949; for more recent discussions, see Matthen, 2010; Fernández, 2019; Rosen and Barkasi, 2021): not only are the events experienced as past, as something that took place in the past, but they are also experienced as part of their personal past,^3^ which often leads to a re-experience. The experience of reliving and reexperiencing the past from a third-person perspective is expected to be common in these cases, given that in a block-universe, events at a given time are fixed and unchangeable. It is worth noting that although the possibility of altering the past or certain aspects of it exists in some theories of time, such possibility is generally considered highly unlikely. More orthodox theories of time posit that the past can be influenced by our actions but cannot be changed (see section 8 for more details). In these cases, the time traveler would not relive the totality of past events, but by affecting the past (without changing it), they would also create new experiences and events that were never previously lived.

In consequence, there are three distinct types of conscious experiences that the time traveler can undergo: (a) a completely new experience, (b) a complete re-experience, but from a third-person (visual) perspective, and (c) a mixed experience where some aspects are re-experienced from a third-person (visual) perspective while others are experienced as new. All these experiences come with a feeling of presence, given that the past is *physically* present, both temporally and spatially. At the same time, a feeling of pastness can be also part of the experience, specially in (b) cases, where what is present is experienced as some events that already took place in our personal past.

Cases falling under category (a) appear to be incompatible with MTT, particularly those that do not exhibit even a minimal sense of pastness. While some philosophers argue that the mere activation of a memory trace is sufficient for a mental state to be considered a memory, allowing us to remember without being aware that we are remembering and perceiving the memory as a new idea generated by our imagination (as seen in the famous painter’s case described by Martin and Deutscher, 1966), these instances, even if deemed genuine memories, do not exhibit any phenomenal features commonly associated with MTT. Unlike TT, primarily characterized by a feeling of presence, the basic feeling that defines MTT is a feeling of pastness in the strong sense introduced earlier: the mental state must, at the very least, feel like something from *my personal* past. Although not sufficient on its own to characterize a mental state as an MTT, as explained below, a memory that loses this feeling may still be considered a memory, but it is experienced more as imagination (Rosen and Barkasi, 2021), rendering the experience of MTT impossible. On the other hand, cases falling under category (a) that could potentially present a minimal sense of pastness, such as imagining a past event not presented as experienced firsthand, like the First World War, also pose challenges for MTT. These events are not experienced as part of our subjective time and lack self-referential qualities, rendering them inherently incompatible with MTT. They can only be recreated through imagination. Although not experienced as part of our subjective time, and thus incompatible with MTT by definition, contemplating the possibility of mentally traveling there is also problematic. Recreating a historical event through imagination is unlikely to present the phenomenal characteristics and evoke even a minimal sense of pastness that are necessary to conceive the possibility of “traveling back” (see also the discussion about “episodic counterfactual thinking” at the end of this section). Empirical research is required to take a stance on this matter, and individuals with hyperphantasia, characterized by their ability to entertain imagery “as vivid as real seeing” (Zeman et al., 2020), might be potential candidates for mentally traveling to past times they know they did not personally experience and do not feel as if previously experienced. However, in principle, this possibility is not compatible with MTT by definition. And even if subjective time were not a requirement for MTT, it is highly unlikely that individuals who are not hyperphantasic could vividly recreate a past they know they did not experience first-hand to the extent of feeling like they are traveling back and reliving those moments. Hence, while cases like (a) are possible in TT, they are likely to be incompatible with MTT.

Cases falling under category (b) represent the most commonly observed phenomenology associated with MTT in the literature. The events feel like they belong to the past, to my personal past, and they seem as if they were being re-experienced. Although the feeling of pastness, in a strong sense, is the fundamental requirement for MTT, it is not sufficient. A feeling of pastness, in the strong sense, can be part of a feeling of familiarity—a context-free recognition of the prior occurrence of an event in our personal past, without recollection of any contextual details (Mandler, 1980). Simply recognizing that something was previously seen or experienced is not sufficient for the past experience itself to emerge, nor for us to experience traveling there. As Tulving wrote, referring to the classical serial recall tasks, “in order for the subject to actually remember that he saw, or did not see, a test item in the study list he must ‘travel back’ to the study episode” (Tulving, 2002a, p. 18). The re-experience of the episode, which includes the recollection of its contextual details, seems to be necessary to be able to travel back there.

One question that can be asked is how this feeling of re-experience emerges. Numerous researchers have argued that the presence of vivid visual imagery accompanying memory is primarily responsible for the sensation of re-experiencing or reliving, and serves as the most reliable predictor of such experiences. Empirical studies have consistently demonstrated a strong association between personal memories characterized by a heightened sense of reliving and the presence of vivid visual images (Rubin et al., 2003; Greenberg and Knowlton, 2014; Aydin, 2018; Palombo et al., 2018), or “mental scenes” according to the terminology introduced by Rubin and Umanath (2015) and Rubin et al. (2019). Additionally, the format of imagery may contribute to the degree of re-experiencing: Irish et al. (2011) demonstrated that recalling an event with a sense of continuity and a three-dimensional quality, akin to watching a video, was associated with a stronger sense of reliving compared to static visual snapshots resembling photographs. The essential link between visual imagery, the feeling of re-experiencing, and personal memories is further supported by research in psychopathology. For instance, a rare condition known as long-term visual memory loss has been found to give rise to a form of autobiographical amnesia that can be as severe as amnesias caused by extensive damage to the medial temporal lobe (Greenberg et al., 2005; Rubin et al., 2019). Individuals who report a lack of visual imagery, referred to as aphantasia, may still be capable of remembering past experiences but are likely to exhibit deficiencies in autobiographical remembering (Zeman et al., 2010, 2015, 2020). Blind individuals, who typically exhibit lower levels of visual and spatial imagery, also tend to recall fewer memories compared to sighted individuals (Greenberg et al., 2005; Tekcan et al., 2015). Moreover, certain theories of PTSD propose that visual imagery may be responsible for the intense feeling of re-experiencing due to its potent influence on emotional systems (Brewin and Holmes, 2003; Holmes and Mathews, 2010), although it should be noted that some researchers argue that reliving and emotional reliving are not necessarily linked to the traumatic aspect of traumatic memories, but rather associated with their involuntary nature (Rubin et al., 2008). As discussed in section 3, the intensity of these experiences can be so profound that individuals with PTSD may process the traumatic event being remembered as if it were occurring again in the present moment. This involves a momentary deletion of the feeling of pastness, which is then substituted by a feeling of “nowness” (Ehlers et al., 2004; Hackmann et al., 2004; Brewin, 2015) or, in philosophical terminology, a feeling of presence (Nanay, 2016; Rosen and Barkasi, 2021).^4^

Visual imagery has played, and continues to play, a significant role in explaining the feeling of re-experiencing. However, the relationship between visual imagery and emotion, particularly explicit in some theories of post-traumatic stress disorder (PTSD), suggests that this explanation is more complex. In fact, the feeling of reliving can be better understood as a combination of various essential factors that interact with one another, including visual imagery, affective and emotional processes, and self-referential processes (Conway and Pleydell-Pearce, 2000; Rubin et al., 2003; Palombo et al., 2018). Moreover, research has shown that the emotional intensity during the recall of autobiographical memories is correlated with the degree of reliving experienced during the recall (Talarico et al., 2004). On the other hand, the significance of self-referential processes has recently been emphasized by D’Argembeau (2020), who suggests that event simulations need to be integrated with autobiographical knowledge and situated on a personal timeline order to provide a feeling of pastness, which can be considered as a prerequisite for the possibility of re-experiencing the past.

Furthermore, research conducted predominantly in the past two decades has revealed additional correlations between specific types of cues and a heightened sense of reliving the past. While the influence of each of the five senses on the phenomenal aspects of autobiographical memories is still being investigated (Ernst et al., 2021), auditory cues, particularly music, and odor cues appear to elicit stronger feelings of reexperiencing and enhanced memory vividness (El Haj et al., 2018). Autobiographical memories triggered by music are characterized by richer episodic details compared to memories evoked by other cues, such as visual images of well-known faces, and are associated with higher self-reported ratings of memory vividness (Belfi et al., 2016). Similarly, odor-evoked memories are accompanied by a more pronounced sense of being transported back in time and reliving the experience in comparison to memories triggered by verbal cues (Willander and Larsson, 2007), visual cues (Herz and Schooler, 2002), and memories recalled without the presence of odor, particularly among individuals with Alzheimer’s disease (Glachet and El Haj, 2019).

Contextual and material factors are also likely to have a significant impact on generating and intensifying the feeling of re-experiencing. The method of “context reinstatement” is commonly employed in eyewitness research due to consistent findings indicating that it enhances memory retrieval by maximizing the similarity between the original context in which an event was experienced and encoded, and the conditions during recall (known as the Encoding Specificity Principle: Tulving and Thomson, 1973; see also Hershkowitz et al., 2002). Context reinstatement can be physical when individuals are exposed to the actual environment where the remembered event took place and was initially encoded. However, it can also be mental when it is achieved through techniques that guide individuals to mentally recreate the environmental context. This involves visualizing the setting, considering other sensory cues such as smells, and taking into account their psychological states during that past time, including mood and emotion (Hershkowitz et al., 2002; Smith, 2013). Existing studies have focused on examining the effect of context reinstatement on the quantity and quality of the information recalled, rather than the phenomenology of the reinstatement itself. Although further research is required, it could be hypothesized that context reinstatement might generally elicit a strong sense of reliving. In fact, some evidence suggests that mental context reinstatement may also exhibit phenomenal characteristics associated with MTT in certain individuals. These include the feeling of mentally traveling back in time and the sensation of re-experiencing the event, which are positively associated with the accuracy of the remembered information (Smith-Spark et al., 2017; Bangs and Smith-Spark, 2020).

The potential of context reinstatement is also implicitly recognized in recent studies and initiatives aimed at reducing the symptoms of people with dementia and Alzheimer’s disease. Miles et al. (2013) demonstrated that older adults with dementia exhibited improved autobiographical remembering when placed in a historically authentic environment that recreated the material and cultural context of their youth, compared to being in a modern setting. Similarly, a study involving an immersive experience at the House of Memories in Denmark—a museum designed to resemble a typical 1950s home, filled with objects from that decade—found that memories of individuals with mild and moderate Alzheimer’s disease, in response to those objects, became more specific, detailed (including thoughts, emotions, and images), and elaborate compared to the memories prior to the intervention (Kirk et al., 2019). However, these studies primarily focus on enhancing the recalled memories themselves rather than their phenomenal aspects. This is likely because much of the research involves elderly adults and individuals with Alzheimer’s and dementia, who may access fragments of their past in a more semantized manner. While further research is needed before drawing broader conclusions, these findings suggest that if imagery and emotional content are correlated with the feeling of reliving, then by extension, physical context reinstatement and the manipulation of past objects and material traces would also be associated with a heightened sense of reliving. This effect may be particularly pronounced in younger adults and non-pathological populations, who generally have less semantized memories compared to older adults (Piolino et al., 2006, 2009; Trakas, 2019a).

The memory enhancement associated with environmental and object cues may not be solely explained by the Encoding Specificity Principle (Tulving and Thomson, 1973). It has been suggested that the multimodal nature of immersive environments and objects, engaging multiple senses such as vision, olfaction, audition, and somatic sensation, activates larger neural networks. This activation includes implicit and procedural memory systems, reducing the reliance on executive control processes and voluntary retrieval. As a result, access to past memories becomes more involuntary and spontaneous (Berntsen et al., 2013; Miles et al., 2013; Kirk and Berntsen, 2018). Involuntary memories, which are more likely to be triggered in these contexts, are characterized by a stronger sense of re-experiencing the past rather than the effortful mental reconstruction often associated with voluntary memories.^5^ In fact, some evidence suggests that involuntary memories tend to be more specific, sensory vivid, and detailed, and elicit a higher sense of reliving compared to voluntary memories. Therefore, factors that promote involuntary memories would also contribute to the feeling of re-experiencing the past (Finnbogadóttir and Berntsen, 2011).

These same explanations could also account for the influence of bodily movements and manipulations in memory processes. There is substantial evidence from studies on eye movements, co-speech gestures, body posture, and bodily expression of emotion, indicating that (a) triggering the sensorimotor components originally encoded during recall facilitates memory retrieval, and (b) engaging in a concurrent task that involves the same sensorimotor resources as those activated during encoding interferes with and slows down memory retrieval (for a comprehensive review, see Ianì, 2019). While there is no specific research on the relationship between bodily manipulations and the feeling of reliving, it is highly likely that bodily movements can also modulate the phenomenology of memory. This aligns with the prediction made by Ianì (2019) sensorimotor simulation model (SMM) of memory, which is considered “one of the most compelling future challenges” (Ianì, 2019, p. 1748) in the study of the modulatory effects of body manipulations on memory. The reenactment of bodily movements performed or observed in the past could enhance the sense of traveling back in time and re-experiencing an event not only mentally but also with the entire body (also Trakas, 2021a). Conversely, asking individuals to perform incongruent bodily movements during the recollection of traumatic experiences, different from those associated with the traumatic event, could potentially reduce the sensation of reliving the event (Ianì, 2019).

In conclusion, based on the empirical research discussed earlier regarding visual imagery, emotional processes, music, odors, environmental factors, objects, and bodily movements, it can be inferred that a more immersive and sensory-rich experience of recalling a past event, along with greater reenactment, is likely to result in a stronger feeling of reliving the past (although there may be individual differences in the quantity and quality of mnemonic experiences: Palombo et al., 2018; Trakas, 2022). Further research is needed to validate this prediction and to improve the use of questions and terminology for measuring the phenomenal experience of memory, as conceptual clarity is currently lacking in empirical studies. For instance, while many autobiographical memory questionnaires differentiate between vividness and the sense of reliving or recollection, there is often a mixing of reliving with the feeling of traveling back in time (Boyacioglu and Akfirat, 2015; Berntsen et al., 2019). On the other hand, some proposals explicitly distinguish between reliving and the feeling of going back in time as two separate variables (Rubin et al., 2003; D’Argembeau and Van der Linden, 2006; Arnold et al., 2011; El Haj et al., 2016). The variable of vividness is also problematic, as it is measured through self-reports in some cases (e.g., Jakubowski et al., 2021), calculated using different formulas considering the perceptual details of the memory in other cases (e.g., Belfi et al., 2016), and sometimes used interchangeably with reliving.

While a complete re-experience is commonly associated with MTT, there is a crucial distinction between the re-experience in TT and MTT. In TT, the past is always relived from a third-person visual perspective,^6^ whereas in MTT, the visual perspective can also be from a first-person point of view (Libby and Eibach, 2002). In fact, the first-person perspective has been linked to greater detail, heightened vividness, emotional intensity, and a stronger feeling of reliving compared to the third-person perspective (Berg et al., 2021; Zaman and Russell, 2022). Despite the fact that it should not be assumed that there is *always* a strong association between specific phenomenal properties and the first-person perspective, especially in psychiatric groups, episodes are generally experienced more intensely and vividly when recalled from the same perspective used during encoding, which tends to be the first-person perspective (Zaman and Russell, 2022). Therefore, while the re-experience in TT is associated with the third-person visual perspective, in MTT it exhibits a stronger correlation with the first-person visual perspective. Furthermore, unlike TT, the flexibility of MTT allows for simultaneous perspectives during a single retrieval (Rice and Rubin, 2009), although memories retrieved from multiple perspectives tend to be less vivid than those experienced from a single perspective (Berg et al., 2021: more about perspective in section 6).

Lastly, while a completely new experience is if not possible at least highly unlikely in MTT, a mixed-experience where certain aspects are re-experienced while others are experienced for the first time (referred to as cases c) is indeed, in certain ways, more plausible. First, we can now, through memory, accurately experience something that, in a certain sense, did not exist before (Hacking, 1995). I delve into these cases further in section 8 when discussing the possibility of changing the past, although it is not clear whether the experience of something that did not exist before (understood in this sense) is really felt as a new experience. Second, some cases of episodic counterfactual thinking, involving the imagination of alternative ways in which past personal events could have occurred (Byrne, 2016; Roese and Epstude, 2017; De Brigard and Parikh, 2019), could also be considered as a mixed form of MTT, where some aspects are re-experienced, and others, such as the alternative outcome or choice, are not. Although in principle possible, empirical studies have indicated significant phenomenal differences compared to episodic memory, such as fewer sensory details, worse spatial composition, and less emotional intensity (De Brigard and Giovanello, 2012). While more research is needed before definitive conclusions can be drawn, the immersive and sensory-rich experience that seems to characterize MTT, as previously explained, appears to be absent or very poorly present in episodic counterfactual thinking. Consequently, episodic counterfactual thinking is not a strong candidate for presenting the phenomenal aspects of MTT. Alternative scenarios to past events, although partially felt as part of our subjective time, are unlikely destinations of MTT.

## 6 The traveler and the past self

In general, philosophers acknowledge the possibility of traveling back in time to a period in which one’s earlier self-existed (Miller, 2006). The issue of bilocation or self-visitation has led to different proposed solutions based on the conception of how objects persist through time. Perdurantists argue that the traveler and their past self are two distinct temporal parts of the same person, characterized by different physical and psychological properties (Miller, 2006). On the other hand, endurantists, who believe that an object persists in time by being wholly present at each moment, have also provided solutions by relating the differences between the traveler and their past self to particular properties, such as temporal or spatial properties (Horwich, 1975; Keller and Nelson, 2001; Miller, 2006). Regardless of the theory of persistence through time adopted, it is necessary to recognize that the time traveler and the past self-differ in at least some properties and can always be distinguishable despite being the same person.^7^

Another aspect to consider, assuming that the past self is simply observed by the time traveler, relates to the perspective adopted by the traveler in relation to observing the situation involving their previous self. As it has been already mentioned, the conscious experience of the time traveler has not been discussed in philosophical literature, but some more or less plausible hypotheses can be proposed. From a visuospatial standpoint, it is likely that the traveler adopts a third person perspective. Unless the traveler acquires an unusual ghost-like ability that allows them to occupy the same space as their past self (Carroll, 2011), they perceive their past self and actions from the viewpoint of an external observer. However, perspectives can extend beyond visuospatial aspects and encompass evaluative and affective dimensions. While evaluative and affective perspectives are more complex than visuospatial perspectives (Trakas, 2021b), a similar reductionistic dichotomy is often employed, distinguishing between evaluative and affective first person/field/internal perspectives and third person/observer/external perspectives (Goldie, 2003, 2012; Habermas, 2006, 2019; Sutton, 2010, 2014). In the context of TT, the first person perspective corresponds to the affect, emotions, and evaluations experienced by the actor of the event, in this case, the past self. On the other hand, the third person perspective refers to the affect, emotions, and evaluations experienced by an observer of the event, in this case, the time traveler. As Goldie (2012) explains regarding memory, over time new knowledge, evaluations, and emotions may arise toward a past event, creating a gap between the perspective of the past self who experienced the event and the perspective of the present self who remembers the event. This gap, which can be epistemic, evaluative, and/or emotional, usually leads the traveler to evaluate and feel differently about what happened compared to their past self. The present perspective remains distinct from that of the experiencing protagonist, and the traveler assumes a role more akin to a witness (Laub and Auerhahn, 1993; Habermas, 2006). For instance, something that previously caused embarrassment may now, with the aid of new knowledge, be viewed as an instance of unfair treatment, thereby altering the evaluation of the past situation and potentially giving rise to new emotions, such as anger and resentment. This gap also emerges when personal transformations have occurred in one’s values and beliefs, and the past self’s actions are incongruent with the present self-concept. For example, the traveler may feel ashamed of their former self for enjoying and having fun while engaging in harassing behavior. From this third person evaluative and affective perspective, the traveler may feel alienated from their past self (Schechtman, 2001), perceiving them as a different person, which can evoke a feeling of “not me” (Libby and Eibach, 2002). Whenever a gap exists between the past self and the traveler in TT, despite both perspectives belonging to the same person, they are embodied in different entities: the past self and the time traveler. There are then two different evaluative and affective perspectives of the same event, existing simultaneously while maintaining their individuality by remaining separate in two different temporal parts or versions of the same person. Therefore, even when the traveler’s perspective has shifted, the first person perspective is always present.

It is indeed possible for the time traveler to adopt a first-person perspective, particularly when their perspective remains unchanged since the occurrence of the event. In this scenario, the past beliefs, values, and emotions are recognized as legitimate, establishing a continuous and emotional connection to the past—a form of empathic access to the past (Schechtman, 2001). Additionally, it is possible for the traveler to adopt a different perspective on the past event, such as a third-person emotional perspective, while still experiencing empathy toward their past self or the situation of their past self (Hoffman, 2000). Furthermore, the traveler may be capable of perceiving the past situation from the viewpoint of their past self (Hutto and McGivern, 2016), thereby experiencing a tendency to find themselves in the past condition (Wollheim, 1984) and relive the past inner world of feelings and sensations (Weber, 1914), without necessarily feeling empathy or actually reliving the past emotion. In extreme cases, this inclination may even lead to emotional contagion, resulting in a sort of “pale reflection” of the past self’s emotions (Hatfield et al., 2009; Trakas, 2021b).

At first sight, MTT lends itself more naturally to a first-person perspective, where the mental time traveler *relives* their past self and assumes their previous perspective by mentally traveling back in time, thereby re-experiencing their past emotions and evaluations (see also section 5). The idea of re-experiencing and reliving past personal episodes is the most common conception of MTT and appears repeatedly in both scientific and philosophical literature (Suddendorf and Corballis, 2007; Perrin and Michaelian, 2017). Early twentieth-century French psychologists and philosophers, while discussing the existence of affective memory, described this reliving as ranging from subtle to vivid, and even sometimes bordering on hallucinatory, as the past self momentarily resurfaces within the present self, allowing for the re-experiencing and reenactment of past emotions and feelings (Paulhan, 1902; Ribot, 1907; Weber, 1914). In less hallucinatory scenarios, fusion can still occur, such as when the traveler describes their past experience using the present tense, as if it were an ever-present, timeless experience that has not yet passed (Laub and Auerhahn, 1993; Habermas, 2006). These instances of “fusion” between the past and present self, possible in MTT, represent a notable distinction compared to TT, where such fusion does not seem likely. Even if the traveler in TT adopts the same perspective as their past self and re-experiences the event alongside their past self, they remain distinguishable as they exist in two different temporal parts or versions of the same person.^8^

Despite being less compatible with the association commonly found in the literature between MTT and reliving past experiences, the evaluative and affective third-person perspective can, in principle, also exist in MTT. However, while it may be distinguishable from the previously adopted perspective of the past self in some cases, in many other cases, the third-person perspective can dominate and completely replace the first-person perspective. This occurs when the evaluative and emotional third-person perspective infuses the entire memory, shaping and coloring it to such an extent that the past experience becomes inseparable from this new perspective. In such instances, the mental time traveler does not travel back in subjective time to relive their former self’s enjoyment and amusement while engaging in harassing behavior, only to later transition to a different level and feel ashamed. Instead, they revisit the past event and experience it through their current perspective: they remember a shameful event rather than a joyful or amusing one (Goldie, 2003, 2012: see also section 8). Although the past does not necessarily become completely incomprehensible, changes in beliefs, values, desires, and goals can lead to a loss of connection with one’s past self and the associated phenomenology (Schechtman, 2001).

Indeed, empirical research provides support for these philosophical viewpoints. Several studies have demonstrated that systematic distortions in the recollection of past emotions align with current appraisals (Levine, 1997; Levine et al., 2009; Patihis et al., 2019). In many instances, this overriding occurs as a result of forgetting itself. As past emotions and evaluations become less accessible and fade over time, they become more susceptible to being influenced by current emotions and evaluations of the event. This bias may serve an adaptive purpose by allowing decisions to be guided by recent and relevant emotional experiences (Levine et al., 2009). On other occasions, overriding one’s previous perspective becomes necessary to facilitate effective coping and achieve emotional closure (Levine et al., 2009). In order to attain emotional closure for a past experience, one must arrive at an appropriate evaluation and emotional response that feels fitting to the event, rather than being trapped in a partial, incomplete, or potentially harmful previous perspective (Goldie, 2012). The erasure of the first-person perspective and its substitution with a third-person perspective, which can result in either a more accurate or more distorted recollection of the memory, is a unique feature of MTT and cannot occur in TT. In TT, the first-person perspective cannot be replaced or erased since it is constantly present, embodied in the past self. As elaborated in the next two sections, this phenomenon is connected to a particular malleability of the *mental* past that is absent when considering the past as a region of space–time.

The malleability of mental time also accounts for the diversity of perspectives that can be adopted in MTT, a phenomenon that is more commonly associated with the realm of the mind compared to TT. Research suggests that individuals can experience more than one visual perspective when recalling events (Rice and Rubin, 2009), implying that emotional and evaluative perspectives may exhibit similar flexibility. As noted by Goldie (2012), when we remember something, we can simultaneously embody both our present perspective and the partial perspective we held at the time, perceiving things from both the past and present viewpoints. The transition between perspectives can be attributed to either fast switching perspectives or experiencing them simultaneously, although further empirical research is needed to explore these possibilities (Rice and Rubin, 2009; Sutton, 2014; McCarroll, 2018).

There is one notable distinction between TT and MTT regarding the interaction between visual perspectives and affective/evaluative perspectives. In TT, the traveler’s affective and evaluative perspectives are always coupled with an observer visual perspective (with the exception of the rare case of having ghost-like abilities). In MTT the relationship between types of perspectives is more flexible. While there is generally a correlation between visual perspectives and affective/evaluative perspectives, such as third-person visual perspectives being less emotionally charged than first-person visual perspectives (Robinson and Swanson, 1993; McIsaac and Eich, 2004; Berntsen and Rubin, 2006), and third-person evaluative and affective perspectives being visualized from a third-person perspective with less reliving of past experiences (Libby and Eibach, 2002), these relationships are not always synchronized and can, in principle, vary independently (Sutton, 2010; McCarroll and Sutton, 2017). It has been suggested that individuals can adopt a range of viewpoints, both internal and external, visual and non-visual, that can blend or integrate in different ways when recalling the past (McCarroll and Sutton, 2017; McCarroll, 2018). In fact, incorporating multiple affective and evaluative perspectives, including those of others involved in the past event, may provide the most comprehensive understanding of the past experience (Habermas, 2019; Trakas, 2019b). Further empirical research is nevertheless necessary to fully explore these possibilities.

## 7 The existence of the past

In order to travel through time, there must be distinct temporal parts or stages, situated at various times and places, that exhibit changes between them. The intended destination must have actual existence as a physical location in the space–time manifold (Grey, 1999; Bardon, 2013; Markosian, 2020). Time travel, as depicted in TT, necessitates a Parmenidean world composed of existing events in space–time, which is commonly referred to as the block-universe theory. In a block-universe, all past, present, and future events coexist within a four-dimensional manifold of space–time. Although these events are organized by unchanging relationships, such as being earlier than, later than, or simultaneous with each other, all points in time hold equal ontological status (Miller, 2013). The block-universe theory supposes an eternalist conception of time. It is widely held that other conceptions of time, such as presentism (the idea that only the present moment is real) and the dynamic theory of time (which considers that the present moment changes and thus there are fundamental differences between the present, the past and the future), are incompatible with TT (Grey, 1999; Hales, 2010; Bardon, 2013; Markosian, 2020). As explained by Markosian (2020), even though presentism acknowledges the existence of past and future as past- and future-tensed truths, they do not qualify as travel destinations since they are not physical locations within a manifold but rather logical constructs. Therefore, presentism is generally considered as logically incompatible with TT. Presentism may attempt to make TT intelligible and metaphysically possible (see, for example, Keller and Nelson, 2001; Bernstein, 2022), but still TT may be impossible, in the sense of being physically impossible according to the natural laws of our universe. Furthermore, the growing-block theory of time, which posits that the past and present are fixed and actual while the future remains only possible, may also be incompatible with TT. If TT to the future is not logically possible, then TT to the past may similarly be deemed impossible (Dainton, 2001). Consequently, TT appears to be primarily compatible with eternalism and the block-universe theory.

In MTT, it should also be necessary for the past to have some form of existence in order to travel there, though not necessarily a physical existence in the space–time manifold. This characteristic is important because it is difficult to conceive the notion of “travel,” even in the mental realm, when the destination does not have any kind of possible existence, as previously discussed. An example from Proust illustrates this idea. In his writings, Proust considered that in voluntary recall, the past felt “dead,” and so, nothing of it was really preserved (Proust, 1928, p. 33). There was no destination and no sense of traveling back to it. However, when he tasted a madeleine dipped in tea and ruminating on his memories of Combray, the experience was entirely different. The past suddenly came to life: “and the whole of Combray and its surroundings, taking their proper shapes and growing solid, springing into being, town and gardens alike, from my cup of tea” (Proust, 1928, p. 66).

Involuntary memories, particularly those related to trauma and PTSD, serve as powerful agents that bring the past alive and give it a feeling of existence. Intrusive recollections or flashbacks have been considered to be a “substitute” of the past traumatic event, keeping it “alive” in one’s consciousness (Baum, 1990; Holman and Silver, 1998). The feeling of “nowness” or presence that substitutes the feeling of pastness in some flashbacks may play a role in their intense phenomenology and in their capacity to bring the “past” to life. Similarly, forgotten memories that resurface to consciousness, possibly triggered by external cues, can exhibit this same phenomenal trait. This aligns with the example provided by Paulhan (1902, p. 558) of Émile Littré, a French intellectual who, in his old age, felt a surge of excitement upon recollecting the distant memory of his young sister’s death, an event that had ceased to evoke any emotion in him for a long time. The past resurfaced as if it were brought back to life, even though there was no current interest in it and no active desire or cognitive effort to retrieve it. However, there is an important distinction between MTT and TT in these cases. While there may be a feeling of a past that exists independently of us, the feeling of actively traveling to this existing past is not necessary. The phenomenology of these memories could be better described as an existent past, whether traumatic or forgotten, imposing itself upon us in the present, rather than us consciously embarking on a mental travel to the past. This type of memory experience would only be similar to TT in cases of unplanned and involuntary time travel, which are probably unlikely or less frequent, as discussed in section 3.

Memories that arise through the technique of “context reinstatement” (previously discussed in section 5) are likely a better illustration of memories that can potentially evoke a sense of traveling back to an existing past. When individuals are instructed to mentally place themselves back in the time of a past event they experienced, the feeling of mentally traveling back in time and the sensation of re-experiencing the event are heightened compared to when they are simply asked to recall everything they can remember about the event (Smith-Spark et al., 2017). However, even though memories facilitated by mental context reinstatement may offer a sense of traveling back to an existing past, this past exists solely as a *mental construct*. In this respect, it differs from the type of existence attributed to the past in TT, which requires a physical reality as a location within the four-dimensional manifold of space–time. Thus, this represents a significant distinction between TT and its mental counterpart.

This comparison, however, raises the question of whether in MTT the past can exist beyond the act of remembering. That is, if it can exist not only in a mental form but also in a more permanent and *material* form, albeit not necessarily as a location in the space–time manifold. Past events sometimes leave material and tangible traces in the environment, known as “exograms” (Donald, 1991), which can take on an iconic nature, such as personal photographs, video recordings, documents, and so on. Material traces can also refer to objects that were associated with a past event and have acquired an indexical nature, thus serving as a reference to that event (for instance, a vase broken during a dispute and later repaired; Trakas, 2015; see also Habermas and Paha, 2002). Material traces and exograms can reinstantiate memories. They not only act as cues that facilitate MTT, but they can also become part of the *hybrid* processes of remembering, which integrate biological memory systems with the manipulation and interaction with these material traces (Donald, 1991; Radley and Taylor, 2003; Trakas, 2015; Fawns, 2020; Heersmink, 2022). In these hybrid processes of MTT, the past has more than just a mental existence; it also has a physical, material presence. Photographs and other iconic material traces are likely the best examples. As Nikiforos and Karakitsou (2020) explain, photographs preserve a glimpse of past reality and provide an opportunity to revisit the past “on command,” immersing oneself “in a life-story, embracing the picture’s content as a testament of lived reality” (p. 129). While specific studies focused on the phenomenology of these hybrid or “blended” memories (Fawns, 2020) are missing, users of Sensecam, a wearable digital camera that automatically takes photographs, have anecdotally reported experiencing a “Proustian moment” when viewing particular photographs, where even past thoughts, feelings, and emotions resurfaced (Hodges et al., 2011). The same effect may be present in physical context reinstatement, such as immersive experiences that recreate past material and cultural contexts for dementia patients, as well as any immersive experience in a setting rich with significant objects. Although it is not clear in all these cases whether the experience corresponds to a travel to the past or a past that comes forward to the present, the feeling of moving from the present to a past that exists independently of us may be reinforced when material traces or purposely created replicas from that same past are part of the process of remembering.

Therefore, it can be asserted that in certain cases of MTT, the past possesses not only a mental existence but also a physical one—albeit not as a location within the four-dimensional manifold of space–time, as in TT. In MTT, the past can be physically present through exograms, that is, material records of the past such as photographs, and other material traces with an indexical nature that point to the past. Nevertheless, exograms and material traces are not always necessary to enable the emergence of the mental past. This prompts us to delve deeper into this line of inquiry and question whether the mental existence of the past in MTT necessitates nevertheless other specific physical conditions to arise. For instance, traces of the past can also reside in connections between neurons. Do memory traces or “engrams” (Semon, 1921)—neural changes resulting from the encoding of an experience and stored until retrieval—play a crucial role in the emergence of the mental past? As Tulving (2002a,b) explains: “an event happens, a person experiences it, memory traces are laid down representing the event, the past vanishes and is replaced by the present. The memory traces of the event continue to exist in the present, they are retrieved, and the person remembers the event” (Tulving, 2002a,b, p. 19). Memory traces can then be regarded as the remnants of the past that physically endure in the present through neural connections, enabling the emergence of the “mental” past.

The existence and nature of memory traces, as well as the causal connection to the past, continue to be subjects of controversy (Moscovitch, 2007; Nadel, 2007; Dudai, 2012; Robins, 2017, 2018, 2020a; De Brigard, 2020; Hutto, 2022; Sutton and O’Brien, 2022; De Brigard, 2023). However, even if memories are ultimately rooted in physical, neural memory traces, these traces do not appear to be necessary for explaining the phenomenal experience of mentally traveling back to the past. Therefore, they cannot justify the “mental” existence of a past destination. Phantom recall (Brainerd et al., 2003) and mnemonic confabulation (Robins, 2020b) are examples of memories about events that did not occur in the past (and not about false details), which are “wholly inaccurate, reflecting no influence of information retained from a particular past event” (Robins, 2019, p. 2148). Despite not being grounded in a specific memory trace and exhibiting subtle phenomenal differences under certain circumstances compared to veridical memories, mnemonic confabulations can be phenomenally indistinguishable from genuine memories and can present an extremely rich phenomenology (Lampinen et al., 1997; Jou and Flores, 2013). Not only have empirical studies accounted for this exceptional phenomenal richness, but anecdotal evidence also supports it. For example, Lampinen et al. (1997) highlight the vividness, clarity, and perceptual richness of Piaget (1962) confabulatory memory of a supposed attempted abduction from his childhood, which he recognized as false. The vividness was so intense that Piaget went as far as claiming to be able to re-perceive the event. This phenomenon is not uncommon, as both pathological mnemonic confabulations and non-pathological confabulations, like Piaget’s memory and other non-believed memories, can exhibit this phenomenal richness (Mazzoni et al., 2010; Trakas, 2021c). Similarly, while flashbulb memories could also serve as an example, they may not be the most ideal illustration due to their potential for recalling a different event that actually took place, albeit not at the time of the surprising or shocking news (Jou and Flores, 2013). Therefore, the notion that the mental existence of the past in MTT must ultimately rely on some form of physical existence, such as memory traces in the brain, appears to be unfounded. While physical memory traces often accompany MTT and may influence the phenomenology of memory, such as in cases of traumatic memories associated with PTSD, they do not appear to be essential. MTT can occur phenomenally without the need of physical traces.^9^

In conclusion, whereas in TT the existence of the past is contingent upon its physical manifestation, MTT allows for a past that does not require physical existence. Although exograms and engrams left by the past can contribute to the existence of a past destination in some cases, the past destination can also be a mere mental construct, without the necessity of having a physical existence. The past destination of MTT must—at least—have existed in the past and left an engram to *veridically* remember (Robins, 2020b), but neither its physical existence in space–time nor its present traces are necessary for the act of mentally traveling to it. The past destination in MTT is not bound by conditions of physical existence and is therefore more flexible and malleable compared to the past destination in TT.

## 8 A changing or unchanged past

As mentioned in previous sections, it is widely accepted that only a block-universe framework aligns solely with TT, where every event exists in a fixed and unchangeable state. Consequently, time travelers are unable to alter events that are already unchangeable, which contradicts the common portrayal of altering the past found in science fiction (Horwich, 1975; Lewis, 1976; Grey, 1999; Dainton, 2001; Bardon, 2013; Miller, 2017b). While time travelers cannot change the past, they can certainly interact within it and causally *affect* it through their actions. However, their actions are inherently intertwined with the formation of the past as it always has been: if Tim travels to the past, it is both true and has always been true that his time travel and the actions he undertook occurred. Therefore, the past includes the actions of time travelers. While changing the past may be impossible, the possibility of affecting the past allows for the plausibility of reverse causation, where past events could be caused by future ones.

Alternative models of time, which deviate from the block-universe perspective and are often considered less plausible, have attempted to accommodate a form of TT where the past, or at least certain aspects of it, can in principle be changed. One such approach involves the introduction of additional universes: the traveler goes back to a past situated in a different universe from their point of departure, yet similar enough to the past of their original universe (Dainton, 2001; Effingham, 2023). In this new universe, the traveler can make changes as desired, although some argue that no actual changes occur since their original universe remains unaffected (Miller, 2017b). Rather, they have simply transitioned to another universe or triggered the creation of a new universe upon their arrival. Another option explored in the literature to allow for changing the past in TT involves the introduction of a supplementary temporal dimension known as hypertime. According to Van Inwagen (2010), a time traveler can change the past by relocating to an earlier temporal point, resulting in the annihilation of all events between their departure and arrival. Nevertheless, one can still contend that no genuine change occurs, but rather the emergence of another version of that particular time (Miller, 2017b). Bernstein (2016) theory of the movable objective present (MOP) also incorporates the possibility of change, but these changes manifest in a new present rather than the past. As the time traveler repositions the objective present for the entire temporal manifold, effectively resetting reality, it is the new present that is shaped by the causal results of their actions, rather than the past itself. In conclusion, the notion of changing the past through time travel appears improbable for two reasons: first, it contradicts the most plausible model of space–time, the block-universe theory; and second, in alternative models of time, the apparent changes following TT primarily affect the new present rather than the past itself.

Unlike TT, where changes to the past are considered a highly improbable possibility, MTT operates on the premise that changes to the past are the norm. This aligns with Loftus’ assertion that “all memory is false to some degree” (Berntsen and Loftus, 2009, p. 373), which could be better formulated as “all memory undergoes some degree of change.” Memory inherently involves change, and while certain changes may result in falsehoods, such as source monitoring errors and spontaneous confabulations (Kopelman, 1999), many other changes do not render the memory false.

First and foremost, memories are not literal snapshots of experience preserved in a perceptual form. True memories are always summary representations of our experiences (Conway, 2009), encompassing a level of generalization and abstraction, and thus, change. In MTT, not only general events (a trip to Malaysia) and entire life-time periods (childhood) are summarized and compacted possible destinations (see also Trakas, 2019a), but even experience-near memories, such as flashbacks (which are not exclusive to traumatic events; Berntsen, 2001), are no longer unanimously considered to be conceptually unprocessed and fixed in an unchangeable form. It has been argued that even flashbacks associated with trauma result from highly reconstructive processes, inevitably involving some level of abstraction and condensation influenced by our conceptual knowledge (see, e.g., Berntsen and Rubin, 2008; Berntsen and Nielsen, 2022). As Conway suggests, “episodic information is more summarized and generic, more *representative* of an experience than it is a literal record” (Conway, 2009, p. 2305). Changes in condensation and summarization are inherent in every memory and consequently in every instance of MTT. The act of mentally traveling back in time itself alters the destination by creating a more or less condensed and summarized version of the past. Even if the destination may appear *phenomenally* similar to the past experience, *metaphysically* we always revisit a changed past in MTT—a past that differs from the actual past without necessarily being false. This distinction sets MTT apart from TT, where the prevailing hypothesis posits that, from a metaphysical perspective, the revisited past remains unchanged, always as it was before, even if, phenomenally, some or all of the past events feel new and unfamiliar to the time traveler.

Secondly, there are other types of changes that *certain* memories can undergo without necessarily becoming false. To illustrate this point, a concrete example can be helpful. In her autobiographical book *Le Consentement* (Springora, 2020), Vanessa Springora, a French publisher and writer, recounts her relationship with writer Gabriel Matzneff, which began when she was 14 and he was 49. Initially, she believed he was her true and only love, someone who genuinely cared for her and educated her. The transgression of societal norms felt like freedom, maturity, and a choice to her. However, things changed over time as she gained new experiences and knowledge. She discovered that their relationship was not an exception, as he had many other young lovers (in fact, she met and spoke with one of them). She also developed feelings for another boy, experienced depression and a psychotic episode, and became a mother. Additionally, concepts like “perverse narcissism,” “sexual predator,” and “psychological violence” became more widely recognized and accessible in society, including to her. Social values and morals also shifted, with pedophilia no longer considered an acceptable transgression in the French academic and literary elite, as it was during Springora’s teenage years. These new experiences, knowledge, concepts, and changes in social values led Springora to gradually remember her relationship with Gabriel Matzneff through a completely different lens. She came to realize that she had not been in a loving relationship, but rather a victim of sexual abuse at the hands of a “pervert,” an “ogre,” a “liar,” and a “manipulator” who also held her captive through his books, where he openly described real-life experiences he had with her.

When Springora engages in mental time travel to the period of her relationship with Matzneff, her recollection no longer portrays a loving connection with her one true love. Instead, she remembers an experience characterized by sexual abuse and manipulation at the hands of a pedophile and perverted person. As discussed in section 6, current appraisals and perspectives can override past appraisals and perspectives of the same event (Levine, 1997; Levine et al., 2009). While she may still have access to her former beliefs and emotions toward Matzneff in a propositional form, the actual past event itself, which is the object of her memory and the destination of a mental movement in her personal subjective timeline, has irreversibly changed. It has transitioned from a loving and caring relationship to a case of sexual abuse, mirroring the earlier example of a once amusing instance of bullying that later becomes a shameful event (see section 6; Goldie, 2003, 2012). While this process can sometimes entail distortion and falsehood, such as when depression and present negative mood taint positive or partially positive memories of past events (Urban et al., 2018), in cases like Springora’s, where the past is appraised through newly acquired knowledge, concepts, and personal and social values, this is not the case. When past actions are presented under new concepts and descriptions, new accurate propositions about those actions can be formed in the present without, paradoxically, being true at the time they occurred, since certain knowledge, concepts, and values were either unavailable or different during that period (Hacking, 1995).

Different explanations can account for the emergence of new accurate propositions: either the past remains unchanged while our interpretation or mental representation of it varies, and these new accurate propositions pertain to these interpretations or mental representations of the past; or the past itself has undergone changes, or reality has been reset, and a new version of that particular past has emerged. These are challenging questions to address, as they delve into metaphysical issues and are thus controversial. Due to their nature, a more extensive and profound theoretical discussion is certainly needed; however, some tentative answers can be provided here. The first possibility, is probably the only that is not problematic at all for a block universe theory of time, given that what changes are not the past physical events themselves, but just their present interpretation.^10^ Nonetheless, it relies on certain assumptions about reality and concepts. Reality is exhausted by the series of physical events that occur within it, so if there were an imaginary “sky camcorder” documenting every occurrence in a specific scene, it would be sufficient to capture all that is real (Sharrock and Leudar, 2002). If reality is determinate, then the past is as well: certain events either happened and were experienced, or they did not occur at all. Concepts do not play a role in shaping reality or determining the events themselves. Consequently, the objects of memory are definite and determined, existing independently of memory. A true memory recalls those events as they were experienced, while a false memory involves facts and events that did not take place (see Hacking, 1995). While some may agree with these assumptions and apply them to the physical world, maintaining them becomes somewhat challenging when it comes to the realm of *social* reality.

Although the decomposition of human actions into physical events, such as bodily movements, may be appear determinate, it can be argued that human actions themselves are shaped by concepts: two hands shaking can mean making a deal or saying goodbye (Hacking, 1995); an uprising can signify either a revolution or a coup d’état. Concepts, more specifically social concepts, bring social reality into being. That is why human actions and social reality inherently possess a degree of indeterminacy, which is filled with the understanding and sensibilities of a specific society at a given time, yet remains open to change.^11^ When knowledge, concepts, values and morals change, not only can our memories of the past change, but the past events themselves can be altered, as past *human* events extend beyond mere physical occurrences. It is not merely a matter of revising our opinions and interpretations of past actions; rather, “in a certain logical sense what was done itself is modified” (Hacking, 1995, pp. 249–250). Through the faculty of memory (and additionally, by means of historical research), present-day knowledge, concepts, values and morals shape and determine past human actions and events, either by changing the past itself or by resetting reality and giving rise to a new version of that past. As Hacking stated, “at the very least, we rewrite the past, not because we find out more about it, but because we present actions under new descriptions” (Hacking, 1995, p. 243).

However, there exists a third form of change or reality-reset, somewhat distinct from the previous cases, wherein certain memories can undergo alterations without necessarily becoming false. In the preceding instances, when new concepts and values become socially and personally available, we find more about the past because new descriptions of past human actions become socially available, or available to us. However, in other cases, we find more about the past because *time* is required for the past to take on a more defined shape and be conceived in a more specific manner. This is particularly relevant to certain emotions and feelings. First-order phenomenology (Lambie and Marcel, 2002) or core affective feelings (Russell and Barrett, 1999; Barrett et al., 2007; Russell, 2009; Barrett, 2017) require binding to different concepts and interpretations of the situation and sequence of events that gave rise to them in order to generate a top-down conscious experience of an emotion. While in many cases this binding process occurs simultaneously with the core affective feelings or first-order phenomenology, it does not need to be the case. The ability to be aware of, explicitly identify, and describe one’s feelings may be impaired, known as alexithymia (Hogeveen and Grafman, 2021). An externally-oriented cognitive style that avoids internal and affect-related thoughts can lead to difficulties in identifying or verbally describing feelings, causing a reduction in emotional awareness. Alexithymia is not necessarily pathological but is a dimensional personality trait that varies across the general population, ranging from low to high. In all cases, clinical therapy may be helpful in aiding individuals with alexithymia to effectively articulate their feelings, including past emotions (Cameron et al., 2014).

But there does not necessarily have to be a deficit in the cognitive processing of emotional experiences for a conscious emotional experience to emerge only over time. Certain feelings a person experiences may be too vague when they are fully immersed in the experience itself to take a definite shape and be conceptualized and recognized as, for example, anger during the actual experience. Reflection on the past event may be necessary for the conscious experience of anger to emerge, and therefore, a certain amount of time elapsed from the past event may also be crucial. This characteristic may be particularly applicable to certain emotions, especially higher-order emotions, which can be conceptualized, in some cases, as *trajectory-dependent properties* of (past) events (Jones, 2008). A trajectory-dependent property is a property that applies to states or events based on their position within a broader, structured, and temporally extended context. Ascriptions of trajectory-dependent properties have then temporally extended truthmakers “such that whether it is correct to ascribe a trajectory-dependent property to A at t depends on what happens elsewhen, whether at t + n or at t−n” (Jones, 2008, p. 272). Considering an emotion as a trajectory-dependent property of an event means that the correct attribution of that emotion to the event depends on the trajectory of the event itself. When past events are reevaluated in light of subsequent events and acquire meaning through their contribution to the larger temporal context, not only their significance and impact on our lives becomes contingent on their interrelations with other events, but also the emotions experienced both then and now. Certain emotions can only be revealed or come into existence in the present when the event that elicited them is considered within a broader structure. Consequently, these emotions, now conceptualized as such in the present, can be faithfully attributed to the past event (see Trakas, 2021b for a detailed analysis of the relationship between memory, affectivity and emotion). In contrast to the aforementioned examples, the changes or resetting of the past reality—understood in its full sense, and not merely as physical occurrences—do not stem from changes or the newfound accessibility of concepts and values that were previously absent. Instead, in these latter cases, the past itself, particularly certain affective and emotional aspects, comes into existence in the present through the process of memory. Not only do memories not become false, but they also seem to become more accurate by introducing more determination to previously indeterminate aspects of the human affective and social past.

## 9 Conclusion

As Draaisma (2000) has articulated it, language associated with memory has “a metaphorical cast” (Draaisma, 2000, p. 3). It is not only customary in science to seek understanding of a phenomenon by drawing parallels to concepts that are better understood or concepts that are more familiar (Roediger, 1980) but, particularly in the case of memory, there has been a historical propensity to elucidate this capacity through the use of metaphorical terminology (Roediger, 1980; Draaisma, 2000; Danziger, 2008). While some memory metaphors have been relatively casual comparisons, with no impact on the scientific domain, others have represented serious attempts to formulate models and theories of memory (Roediger, 1980). They have evolved into *literary-scientific constructs* (Draaisma, 2000) that reflect the interests of their authors, the cultural influences, and/or the period and intellectual atmosphere of the time, at the same time that they forge their own perspective of memory, influencing and guiding scientific research (and potentially molding folk conceptualizations of memory).

The memory metaphor of MTT can be regarded as one of such literary-scientific constructs. It has wielded and continues to wield a significant impact on scientific research, prompting us to conceptualize memory as a form of TT that happens *in the mind*. Hence, it is crucial to gain a deeper understanding of what it truly entails to regard memory as a *mental* form of TT. Only by comprehending the complete scope of this metaphor and its implications for the conceptualization of memory can we thoroughly assess its suitability in explaining this phenomenon. The primary aim of this paper was then to provide a detailed characterization of the phenomenon of mental time travel (MTT) to the past. For this purpose, I draw a comparative analysis with time travel (TT) to the past, a concept, although not better understood than memory, that is an older and more familiar notion widely explored in academic research. This analysis has contributed to a more refined characterization of MTT, uncovering significant differences between the two concepts that shed light on the unique nature of MTT.

From a metaphysical perspective, MTT exhibits greater flexibility and malleability compared to TT. The destinations in MTT are not equal to the original past event or experience but undergo processes of abstraction and generalization, allowing for fluctuations and mobility within a single MTT experience. Notably, MTT can even lead to destinations that do not exist and have not existed in spacetime, a feature absent in TT. Unlike TT, which requires physical reality for destinations, MTT thrives as a mental construct, and although memory traces or material traces may be involved in its instantiation in certain cases, MTT seems to be independent of specific physical, material traces of the past. Moreover, in TT, the past remains consistent and unaltered as it is revisited, as it remains unchanged. In MTT, the past encountered is always distinct and sculptured by the process of MTT itself. MTT inherently involves abstractions, generalizations, and, in certain instances, new knowledge, concepts, values, or the mere passage of time, contribute to shaping the unique nature of the revisited past.

Regarding the conscious experience of the time traveler, it can be argued that it exhibits more flexibility and diversity in the case of MTT compared to TT. While TT is generally associated with a third person (visual) perspective and a feeling of presence, MTT is most commonly linked to a first person perspective and a feeling of pastness. Although both perspectives (at least affective and evaluative perspectives) are possible in both TT and MTT, notable differences remain. In MTT, the possibility of experiencing an entirely new event that is not felt as part of our personal past does not seem viable, and re-experiences may also differ, as fusion with the past self is solely possible in MTT. Furthermore, first person perspectives can be overridden and erased by new perspectives only in MTT, and it is not uncommon to encounter multiple and changing perspectives.

To conclude, while MTT and TT to the past share some common aspects, notable distinctions exist. The mental nature of time travel in MTT fundamentally alters its essence, emphasizing the importance of understanding its true aspect to assess its relevance in explaining memory. My aim here was not to judge the adequacy of the MTT metaphor but rather to offer a comprehensive and meticulous characterization of MTT to the past. Such an endeavor may appear modest or inconsequential to some, but it serves as a crucial step in comprehending the MTT metaphor. Additionally, this analysis has brought together a wealth of empirical literature on memory, especially focusing on its phenomenal aspects. This not only enhances our understanding of the conscious experience of MTT but also delves into the metaphysical underpinnings of MTT. In short, my exploration has illuminated the intriguing realm of MTT to the past, revealing its distinctive attributes and encouraging further investigations into the intricacies of this fascinating memory metaphor.

## Data availability statement

The original contributions presented in the study are included in the article, further inquiries can be directed to the corresponding author.

## Author contributions

MT: Conceptualization, Funding acquisition, Investigation, Writing – original draft, Writing – review & editing.

## Footnotes

^1^While an explicit reference to mental time travel is only found in Tulving (1985), the idea that memory involves mentally traveling back in time is already mentioned in his earlier work, *Elements of Episodic Memory* (Tulving, 1983): “Other members of the animal kingdom can learn, benefit from experience, acquire the ability to adjust and adapt, to solve problems and make decisions, but they cannot travel back into the past in their own minds” (p. 1).

^2^I diverge from Byrne (2010) and Matthen (2010) on this point: “If I literally travelled back in time to yesterday’s lunch, I would not only have an experience that felt that it is about the present: in fact, it would be about the present.” (Matthen (2010), pp. 8–9). While a sense of presence of events, objects, and people will be a constitutive part of my experience, a feeling of pastness, akin to what we experience during recollection, can also characterize the traveler’s experience, as I show next. Yesterday’s lunch would be something present but that I have already experienced, so it would feel as something past, more specifically, as something from my personal past. As mentioned earlier, even when the traveler journeys to a period where they lacked firsthand experience, a certain feeling of pastness in a minimal sense can emerge. If I were to return to a period I did not experience firsthand but have some semantic knowledge of, I would likely feel the past when observing people, artifacts, technology, architecture, and other elements of that time, as well as events that I indirectly know through historical science.

^3^The "feeling of pastness" is a philosophical concept frequently recognized as the defining characteristic of episodic memory. Initially introduced, at least in contemporary discussions, by Russell (1921), its nature remains a subject of ongoing debate. I refrain from taking a specific stance on its nature, though I emphasize that it pertains to the feeling that a particular experience occurred in *my personal past* and not merely in the *past*, as defined, for example, by Rosen and Barkasi (2021, p. 98). My conceptualization more accurately captures the essence of the feeling of pastness. So, this is what I refer to as the strong sense of the feeling of pastness, while leaving open the possibility that a certain minimal feeling of pastness can emerge from a simple experience whose content refers to a past event not directly witnessed either as a participant or an observer, such as, for example, thinking about the First World War.

^4^These specific instances of flashbacks, which feel as if they were happening again, contradict the general notion that a feeling of pastness is necessary for MTT. However, due to their infrequency, they do not pose a substantial challenge to this general idea, which applies to the majority of MTT cases, including flashbacks that retain their feeling of pastness. A thorough analysis, beyond the scope of this discussion, is nevertheless required. In principle, the inappropriate feeling of “nowness” or presence that has replaced the appropriate—and expected—feeling of pastness could potentially explain the sensation of traveling back in time and re-living, in a profound sense, a traumatic episode from the past.

^5^Interestingly, in certain studies, a modified version of the Memory Characteristics Questionnaire includes an item that assesses the effort required to recall an event: “the effort required to bring the event to mind” or “how much effort was needed to recall the event.” This item is believed to contribute to the overall subjective reliving scale, with a higher score indicating a greater level of phenomenal experience of MTT (Arnold et al., 2011; Smith-Spark et al., 2017; Bangs and Smith-Spark, 2020). However, the empirical foundation for the correlation between high effort and a heightened sense of reliving in MTT is questionable. A high level of effort to retrieve a memory aligns more with the experience of (re) constructing the past or engaging in a detective-like process, rather than the experience of traveling back in time and reliving the past as a passive observer. This wording of the question differs from others, such as “how difficult it was to travel into the past and future” (Hartmann et al., 2014), where the experience of MTT is implicitly assumed, and the effort measured pertains to the act of traveling back in time rather than the effort involved in memory retrieval.

^6^Unless there is a unique circumstance in which fusion with the past self occurs after traveling back in time, or if the time traveler gains a ghost-like ability to coexist with their past self (for the latter option, see Carroll, 2011 and the following footnote), the first-person visual perspective in time travel is highly improbable and is unlikely to be considered a typical instance of time travel.

^7^Some authors have proposed more complex scenarios to explore potential solutions to the bilocation problem, particularly within an endurantist framework. Miller (2006) presents the idea of a time machine that also reverses the age of the traveler: upon arriving at t−1, the traveler becomes a qualitative duplicate of their past self at t−1. As mentioned in the previous footnote, Carroll (2011) introduces an esoteric scenario where the traveler, after traveling back in time, acquires a ghost-like ability and can coexist in the same space as their past self. However, these scenarios involve additional assumptions beyond the scope of traditional time travel (TT) and are presented as thought experiments to evaluate different theses. For the purposes of this paper, I will omit these highly hypothetical and unlikely scenarios, even within the context of TT, and focus on the more traditional and widely accepted understanding of TT.

^8^This holds true except in the exceptional scenario where the traveler gains a ghost-like ability, as depicted by Carroll (2011).

^9^While the evocation of the mental past undoubtedly requires a physical substrate, such as a body, a human brain, and neural correlates, the crucial question is whether the past events must persist in a specific physical form, like a memory trace, for the mental traveler to recreate and journey to them. As I have argued, it appears that this is not a necessary condition.

^10^This raises potential complications, such as the question of whether we can make true predications about the past when those predications are based solely on our present interpretations. Conversely, a block universe theory of time might allow for the acceptance of changes in past reality originating from conceptual shifts, given that they do not alter the physical events themselves. These questions are intriguing and warrant careful, detailed examination, though a comprehensive answer is beyond the scope of this discussion.

^11^In this context, the idea of “open to change” implies that, given the inherent indeterminacy of social reality, subsequent events may be necessary to determine it (Hacking, 2003).

## References

[ref1] Addis D. R. (2018). Are episodic memories special? On the sameness of remembered and imagined event simulation. J. R. Soc. N. Z. 48, 64–88. doi: 10.1080/03036758.2018.1439071

[ref2] Addis D. R. (2020). Mental time travel? A neurocognitive model of event simulation. Rev. Philos. Psychol. 11, 233–259. doi: 10.1007/s13164-020-00470-0

[ref4] Allman M. J. Teki S. Griffiths T. D. Meck W. H. (2014). Properties of the internal clock: first-and second-order principles of subjective time. Annu. Rev. Psychol. 65, 743–771. doi: 10.1146/annurev-psych-010213-115117, PMID: 24050187

[ref5] Andonovski N. (2021). Memory as triage: facing up to the hard question of memory. Rev. Philos. Psychol. 12, 227–256. doi: 10.1007/s13164-020-00514-5

[ref6] Arnold K. M. McDermott K. B. Szpunar K. K. (2011). Individual differences in time perspective predict autonoetic experience. Conscious. Cogn. 20, 712–719. doi: 10.1016/j.concog.2011.03.006, PMID: 21450493

[ref7] Aydin C. (2018). The differential contributions of visual imagery constructs on autobiographical thinking. Memory 26, 189–200. doi: 10.1080/09658211.2017.1340483, PMID: 29295687

[ref8] Bangs K. Smith-Spark J. H. (2020). Mental reinstatement of context: do individual differences in mental time travel and eyewitness occupation influence eyewitness performance over different delay intervals? J. Investig. Psychol. Offender Profiling 17, 31–45. doi: 10.1002/jip.1536

[ref9] Bardon A. (2013). A brief history of the philosophy of time. Oxford: Oxford University Press.

[ref10] Barkasi M. Rosen M. G. (2020). Is mental time travel real time travel? Philos. Mind Sci. 1, 1–27. doi: 10.33735/phimisci.2020.1.28

[ref11] Barrett L. F. (2017). How emotions are made: the secret life of the brain. New York: Pan Macmillan.

[ref12] Barrett L. F. Mesquita B. Ochsner K. N. Gross J. J. (2007). The experience of emotion. Annu. Rev. Psychol. 58, 373–403. doi: 10.1146/annurev.psych.58.110405.085709, PMID: 17002554 PMC1934613

[ref13] Barsalou L. W. (1988). “The content and organization of autobiographical memories” in Remembering reconsidered: ecological and traditional approaches to the study of memory. eds. Neisser U. Winograd E. (New York: Cambridge University Press), 193–243.

[ref14] Baum A. (1990). Stress, intrusive imagery, and chronic distress. Health Psychol. 9, 653–675. doi: 10.1037/0278-6133.9.6.653 2286178

[ref15] Belfi A. M. Karlan B. Tranel D. (2016). Music evokes vivid autobiographical memories. Memory 24, 979–989. doi: 10.1080/09658211.2015.1061012, PMID: 26259098

[ref16] Berg J. J. Gilmore A. W. Shaffer R. A. McDermott K. B. (2021). The stability of visual perspective and vividness during mental time travel. Conscious. Cogn. 92:103116. doi: 10.1016/j.concog.2021.103116, PMID: 34038829

[ref17] Bernstein S. (2016). “Time travel and the movable present” in Being, freedom, and method: themes from the philosophy of Peter van Inwagen. ed. Keller J. A. (New York: Oxford University Press), 80–92.

[ref18] Bernstein S. (2022). “Paradoxes of time travel to the future” in Perspectives on the philosophy of David K. Lewis. eds. Beebee H. Fisher A. R. J. (Oxford University Press), 241–259.

[ref19] Berntsen D. (2001). Involuntary memories of emotional events: do memories of traumas and extremely happy events differ? Appl. Cogn. Psychol. 15, S135–S158. doi: 10.1002/acp.838

[ref20] Berntsen D. (2009). Involuntary autobiographical memories: an introduction to the unbidden past: Cambridge University Press.

[ref21] Berntsen D. (2021). Involuntary autobiographical memories and their relation to other forms of spontaneous thoughts. Philos. Trans. R. Soc. B 376:20190693. doi: 10.1098/rstb.2019.0693, PMID: 33308074 PMC7741080

[ref22] Berntsen D. Hoyle R. H. Rubin D. C. (2019). The autobiographical recollection test (ART): a measure of individual differences in autobiographical memory. J. Appl. Res. Mem. Cogn. 8, 305–318. doi: 10.1037/h0101839, PMID: 31700775 PMC6836687

[ref23] Berntsen D. Jacobsen A. S. (2008). Involuntary (spontaneous) mental time travel into the past and future. Conscious. Cogn. 17, 1093–1104. doi: 10.1016/j.concog.2008.03.001, PMID: 18424178

[ref24] Berntsen D. M. Loftus E. F. (2009). How to tell if a particular memory is true or false. Perspect. Psychol. Sci. 4, 370–374. doi: 10.1111/j.1745-6924.2009.01140.x 26158984

[ref25] Berntsen D. Nielsen N. P. (2022). The reconstructive nature of involuntary autobiographical memories. Memory 30, 31–36. doi: 10.1080/09658211.2021.1872645, PMID: 33459150

[ref26] Berntsen D. Rubin D. (2006). Emotion and vantage point in autobiographical memory. Cognit. Emot. 20, 1193–1215. doi: 10.1080/02699930500371190

[ref27] Berntsen D. Rubin D. C. (2008). The reappearance hypothesis revisited: recurrent involuntary memories after traumatic events and in everyday life. Mem. Cogn. 36, 449–460. doi: 10.3758/MC.36.2.449, PMID: 18426073 PMC3044895

[ref28] Berntsen D. Staugaard S. R. Sørensen L. M. T. (2013). Why am I remembering this now? Predicting the occurrence of involuntary (spontaneous) episodic memories. J. Exp. Psychol. 142, 426–444. doi: 10.1037/a0029128 22746701

[ref29] Bigelow J. (1996). Presentism and properties. Philos. Perspect. 30, 35–52. doi: 10.2307/2216235

[ref30] Boyacioglu I. Akfirat S. (2015). Development and psychometric properties of a new measure for memory phenomenology: the autobiographical memory characteristics questionnaire. Memory 23, 1070–1092. doi: 10.1080/09658211.2014.953960, PMID: 25202835

[ref31] Brainerd C. J. Payne D. G. Wright R. Reyna V. F. (2003). Phantom recall. J. Mem. Lang. 48, 445–467. doi: 10.1016/S0749-596X(02)00501-6

[ref32] Brewin C. R. (2015). Re-experiencing traumatic events in PTSD: new avenues in research on intrusive memories and flashbacks. Eur. J. Psychotraumatol. 6:27180. doi: 10.3402/ejpt.v6.27180, PMID: 25994019 PMC4439411

[ref33] Brewin C. Holmes E. (2003). Psychological theories of posttraumatic stress disorder. Clin. Psychol. Rev. 23, 339–376. doi: 10.1016/s0272-7358(03)00033-3 12729677

[ref34] Broad C. D. (1925). The mind and its place in nature. New York: Kegan Paul, Trench, Trubner & Co.

[ref36] Byrne A. (2010). Recollection, perception, imagination. Philos. Stud. 148, 15–26. doi: 10.1007/s11098-010-9508-1

[ref37] Byrne R. M. (2016). Counterfactual thought. Annu. Rev. Psychol. 67, 135–157. doi: 10.1146/annurev-psych-122414-033249 26393873

[ref38] Cameron K. Ogrodniczuk J. Hadjipavlou G. (2014). Changes in alexithymia following psychological intervention: a review. Harv. Rev. Psychiatry 22, 162–178. doi: 10.1097/HRP.0000000000000036 24736520

[ref39] Carroll J. W. (2011). Self visitation, traveler time, and compatible properties. Can. J. Philos. 41, 359–370. doi: 10.1353/cjp.2011.0025

[ref40] Conway M. A. (2009). Episodic memories. Neuropsychologia 47, 2305–2313. doi: 10.1016/j.neuropsychologia.2009.02.003 19524094

[ref41] Conway M. A. Pleydell-Pearce C. W. (2000). The construction of autobiographical memories in the self-memory system. Psychol. Rev. 107, 261–288. doi: 10.1037/0033-295X.107.2.261, PMID: 10789197

[ref42] D’Argembeau A. (2020). Zooming in and out on one’s life: autobiographical representations at multiple time scales. J. Cogn. Neurosci. 32, 2037–2055. doi: 10.1162/jocn_a_01556, PMID: 32163320

[ref43] D’Argembeau A. Van der Linden M. (2006). Individual differences in the phenomenology of mental time travel: the effect of vivid visual imagery and emotion regulation strategies. Conscious. Cogn. 15, 342–350. doi: 10.1016/j.concog.2005.09.001 16230028

[ref44] Dainton B. (2001). Time and space. Durham: Acumen.

[ref45] Danziger K. (2008). Marking the mind: a history of memory. New York: Cambridge University Press.

[ref46] De Brigard F. (2020). The explanatory indispensability of memory traces. Harv. Rev. Philos. 27, 23–47. doi: 10.5840/harvardreview202072328

[ref47] De Brigard F. (2023). “Simulationism and memory traces” in Memory, space and time. eds. Aronowitz S. Nadel L. (OUP).

[ref48] De Brigard F. Giovanello K. S. (2012). Influence of outcome valence in the subjective experience of episodic past, future and counterfactual thinking. Conscious. Cogn. 21, 1085–1096. doi: 10.1016/j.concog.2012.06.007, PMID: 22818200

[ref49] De Brigard F. Parikh N. (2019). Episodic counterfactual thinking. Curr. Dir. Psychol. Sci. 28, 59–66. doi: 10.1177/0963721418806512

[ref50] Debus D. (2014). ‘Mental time travel’: remembering the past, imagining the future, and the particularity of events. Rev. Philos. Psychol. 5, 333–350. doi: 10.1007/s13164-014-0182-7

[ref51] Donald M. (1991). Origins of the modern mind: three stages in the evolution of culture and cognition. United States: Harvard University Press.

[ref52] Draaisma D. (2000). Metaphors of memory: a history of ideas about the mind. Cambridge: Cambridge University Press.

[ref53] Droit-Volet S. (2013). Time perception, emotions and mood disorders. J. Physiol. 107, 255–264. doi: 10.1016/j.jphysparis.2013.03.005 23542546

[ref54] Dudai Y. (2012). The restless engram: consolidations never end. Annu. Rev. Neurosci. 35, 227–247. doi: 10.1146/annurev-neuro-062111-150500, PMID: 22443508

[ref55] Dyke H. (2005). The metaphysics and epistemology of time travel. Think 3, 43–52. doi: 10.1017/S1477175600002050

[ref56] Effingham N. (2023). The metaphysical possibility of time travel fictions. Erkenntnis 88, 1309–1329. doi: 10.1007/s10670-021-00403-y

[ref57] Ehlers A. Hackmann A. Michael T. (2004). Intrusive re-experiencing in post-traumatic stress disorder: phenomenology, theory, and therapy. Memory 12, 403–415. doi: 10.1080/09658210444000025 15487537

[ref58] El Haj M. Gandolphe M. C. Gallouj K. Kapogiannis D. Antoine P. (2018). From nose to memory: the involuntary nature of odor-evoked autobiographical memories in Alzheimer’s disease. Chem. Senses 43, 27–34. doi: 10.1093/chemse/bjx064, PMID: 29040475 PMC5863564

[ref59] El Haj M. Kapogiannis D. Antoine P. (2016). Phenomenological reliving and visual imagery during autobiographical recall in Alzheimer’s disease. J. Alzheimers Dis. 52, 421–431. doi: 10.3233/JAD-151122, PMID: 27003216 PMC5147522

[ref60] Ernst A. Bertrand J. M. Voltzenlogel V. Souchay C. Moulin C. J. (2021). The Proust machine: what a public science event tells us about autobiographical memory and the five senses. Front. Psychol. 11:623910. doi: 10.3389/fpsyg.2020.623910, PMID: 33551934 PMC7854910

[ref61] Fawns T. (2020). Blended memory: a framework for understanding distributed autobiographical remembering with photography. Mem. Stud. 13, 901–916. doi: 10.1177/1750698019829891

[ref62] Fernández J. (2019). Memory: a self-referential account. New York: OUP.

[ref63] Finnbogadóttir H. Berntsen D. (2011). Involuntary and voluntary mental time travel in high and low worriers. Memory 19, 625–640. doi: 10.1080/09658211.2011.595722, PMID: 21919590

[ref64] Gardiner J. M. (2001). Episodic memory and autonoetic consciousness: a first-person approach. Philos. Trans. R. Soc. Lond. B Biol. Sci. 356, 1351–1361. doi: 10.1098/rstb.2001.0955, PMID: 11571027 PMC1088519

[ref65] Glachet O. El Haj M. (2019). Emotional and phenomenological properties of odor-evoked autobiographical memories in Alzheimer’s disease. Brain Sci. 9:135. doi: 10.3390/brainsci9060135, PMID: 31185649 PMC6627121

[ref66] Goldie P. (2003). One's remembered past: narrative thinking, emotion, and the external perspective. Philos. Pap. 32, 301–319. doi: 10.1080/05568640309485129

[ref67] Goldie P. (2012). The mess inside: narrative, emotion, and the mind. New York: Oxford University Press.

[ref68] Greenberg D. L. Eacott M. J. Brechin D. Rubin D. C. (2005). Visual memory loss and autobiographical amnesia: a case study. Neuropsychologia 43, 1493–1502. doi: 10.1016/j.neuropsychologia.2004.12.009, PMID: 15989939

[ref69] Greenberg D. L. Knowlton B. J. (2014). The role of visual imagery in autobiographical memory. Mem. Cogn. 42, 922–934. doi: 10.3758/s13421-014-0402-5 24554279

[ref70] Grey W. (1999). Troubles with time travel. Philosophy 74, 55–70. doi: 10.1017/S0031819199001047

[ref71] Habermas T. (2006). Who speaks? Who looks? Who feels? Point of view in autobiographical narratives. Int. J. Psychoanal. 87, 497–518. doi: 10.1516/AXWM-QRNF-H69K-9K3C, PMID: 16581588

[ref72] Habermas T. (2019). Emotion and narrative: perspectives in autobiographical storytelling. Cambridge: Cambridge University Press.

[ref73] Habermas T. Paha C. (2002). “Souvenirs and other personal objects: reminding of past events and significant others in the transition to university” in Critical advances in reminiscence work. eds. Webster J. D. Haight B. K. (New York, NY: Springer), 123–138.

[ref74] Hacking I. (1995). Rewriting the soul: multiple personality and the sciences of memory. Princeton: Princeton University Press.

[ref75] Hacking I. (2003). Indeterminacy in the past: on the recent discussion of chapter 17 of ‘rewriting the soul’. Hist. Hum. Sci. 16, 117–124. doi: 10.1177/0952695103016002006

[ref76] Hackmann A. Ehlers A. Speckens A. Clark D. M. (2004). Characteristics and content of intrusive memories in PTSD and their changes with treatment. J. Traum. Stress 17, 231–240. doi: 10.1023/B:JOTS.0000029266.88369.fd, PMID: 15253095

[ref77] Hales S. D. (2010). No time travel for presentists. Logos Episteme 1, 353–360. doi: 10.5840/logos-episteme2010129

[ref78] Harris C. B. Keil P. G. Sutton J. Barnier A. J. McIlwain D. J. (2011). We remember, we forget: collaborative remembering in older couples. Discourse Process. 48, 267–303. doi: 10.1080/0163853X.2010.541854

[ref79] Hartmann M. Martarelli C. S. Mast F. W. Stocker K. (2014). Eye movements during mental time travel follow a diagonal line. Conscious. Cogn. 30, 201–209. doi: 10.1016/j.concog.2014.09.007, PMID: 25307523

[ref80] Hatfield E. Rapson R. L. Le Y.-C. L. (2009). “Emotional contagion and empathy” in The social neuroscience of empathy. eds. Decety J. Ickes W. (Boston: MIT Press), 19–30.

[ref81] Hawking S. (2018). Brief answers to the big questions. New York: Bantam.

[ref82] Heersmink R. (2022). Preserving narrative identity for dementia patients: embodiment, active environments, and distributed memory. Neuroethics 15:8. doi: 10.1007/s12152-022-09479-x

[ref83] Hershkowitz I. Orbach Y. Lamb M. E. Sternberg K. J. Horowitz D. (2002). A comparison of mental and physical context reinstatement in forensic interviews with alleged victims of sexual abuse. Appl. Cogn. Psychol. 16, 429–441. doi: 10.1002/acp.804

[ref84] Herz R. S. Schooler J. W. (2002). A naturalistic study of autobiographical memories evoked by olfactory and visual cues: testing the Proustian hypothesis. Am. J. Psychol. 115, 21–32. doi: 10.2307/1423672, PMID: 11868193

[ref85] Hodges S. Berry E. Wood K. (2011). Sense cam: a wearable camera that stimulates and rehabilitates autobiographical memory. Memory 19, 685–696. doi: 10.1080/09658211.2011.605591, PMID: 21995708

[ref86] Hoffman M. L. (2000). Empathy and moral development. Cambridge: Cambridge University Press.

[ref87] Hogeveen J. Grafman J. (2021). “Alexithymia” in Handbook of clinical neurology: disorders of emotion in neurologic disease. eds. Heilman K. M. Nadeau S. E., Vol. 183 (Elsevier), 47–62. 10.1016/B978-0-12-822290-4.00004-9 PMC8456171 34389125

[ref88] Holman E. A. Silver R. C. (1998). Getting "stuck" in the past: temporal orientation and coping with trauma. J. Pers. Soc. Psychol. 74, 1146–1163. doi: 10.1037/0022-3514.74.5.1146 9599436

[ref89] Holmes E. Mathews A. (2010). Mental imagery in emotion and emotional disorders. Clin. Psychol. Rev. 30, 349–362. doi: 10.1016/j.cpr.2010.01.001 20116915

[ref90] Horwich P. (1975). On some alleged paradoxes of time travel. J. Philos. 72, 432–444. doi: 10.2307/2025013

[ref91] Hunter J. B. (2004). “Time travel” in Internet encyclopedia of philosophy. ed. Wrenn C. B.. Available at: http://www.iep.utm.edu/timetrav/

[ref92] Hutto D. D. (2022). “Remembering without a trace? Moving beyond trace minimalism” in Current controversies in philosophy of memory. eds. Sant’Anna A. McCarroll C. J. Michaelian K. (Abingdon: Routledge).

[ref93] Hutto D. D. McGivern P. (2016). “Updating the story of mental time travel: narrating and engaging with our possible pasts and futures” in Time and the philosophy of action. eds. Altshuler R. Sigrist M. J. (Abingdon: Routledge), 141–158.

[ref94] Ianì F. (2019). Embodied memories: reviewing the role of the body in memory processes. Psychon. Bull. Rev. 26, 1747–1766. doi: 10.3758/s13423-019-01674-x, PMID: 31654375

[ref95] Irish M. Lawlor B. A. O'Mara S. M. Coen R. F. (2011). Impaired capacity for autonoetic reliving during autobiographical event recall in mild Alzheimer's disease. Cortex 47, 236–249. doi: 10.1016/j.cortex.2010.01.002, PMID: 20153463

[ref96] Jakubowski K. Belfi A. M. Eerola T. (2021). Phenomenological differences in music-and television-evoked autobiographical memories. Music Percept. 38, 435–455. doi: 10.1525/mp.2021.38.5.435

[ref97] Jeunehomme O. D’Argembeau A. (2019). The time to remember: temporal compression and duration judgements in memory for real-life events. Q. J. Exp. Psychol. 72, 930–942. doi: 10.1177/1747021818773082, PMID: 29649947

[ref98] Jones K. (2008). “How to change the past” in Practical identity and narrative agency. eds. Mackenzie C. Atkins K. (New York: Routledge), 269–288.

[ref99] Jou J. Flores S. (2013). How are false memories distinguishable from true memories in the Deese–Roediger–McDermott paradigm? A review of the findings. Psychol. Res. 77, 671–686. doi: 10.1007/s00426-012-0472-6, PMID: 23266577

[ref100] Keller S. Nelson M. (2001). Presentists should believe in time-travel. Australas. J. Philos. 79, 333–345. doi: 10.1080/713931204

[ref101] Kirk M. Berntsen D. (2018). A short cut to the past: cueing via concrete objects improves autobiographical memory retrieval in Alzheimer's disease patients. Neuropsychologia 110, 113–122. doi: 10.1016/j.neuropsychologia.2017.06.034, PMID: 28676268

[ref102] Kirk M. Rasmussen K. W. Overgaard S. B. Berntsen D. (2019). Five weeks of immersive reminiscence therapy improves autobiographical memory in Alzheimer’s disease. Memory 27, 441–454. doi: 10.1080/09658211.2018.1515960, PMID: 30198380

[ref103] Kopelman M. D. (1999). Varieties of false memory. Cogn. Neuropsychol. 16, 197–214. doi: 10.1080/026432999380762

[ref104] Koriat A. Goldsmith M. (1996). Memory metaphors and the real-life/laboratory controversy: correspondence versus storehouse conceptions of memory. Behav. Brain Sci. 19, 167–188. doi: 10.1017/S0140525X00042114

[ref105] Lambie J. A. Marcel A. J. (2002). Consciousness and the varieties of emotion experience: a theoretical framework. Psychol. Rev. 109, 219–259. doi: 10.1037/0033-295x.109.2.219, PMID: 11990318

[ref106] Lampinen J. M. Neuschatz J. S. Payne D. G. (1997). Memory illusions and consciousness: examining the phenomenology of true and false memories. Curr. Psychol. 16, 181–224. doi: 10.1007/s12144-997-1000-5

[ref107] Laub D. Auerhahn N. C. (1993). Knowing and not knowing massive psychic trauma: forms of traumatic memory. Int. J. Psychoanal. 74, 287–302, 8491533

[ref108] Levine L. J. (1997). Reconstructing memory for emotions. J. Exp. Psychol. Gen. 126, 165–177. doi: 10.1037/0096-3445.126.2.165

[ref109] Levine L. J. Lench H. C. Safer M. A. (2009). Functions of remembering and misremembering emotion. Appl. Cogn. Psychol. 23, 1059–1075. doi: 10.1002/acp.1610

[ref110] Lewis D. (1976). The paradoxes of time travel. Am. Philos. Q. 13, 145–152,

[ref111] Libby L. K. Eibach R. P. (2002). Looking back in time: self-concept change affects visual perspective in autobiographical memory. J. Pers. Soc. Psychol. 82, 167–179. doi: 10.1037/0022-3514.82.2.167, PMID: 11831407

[ref112] Linton M. (1986). “Ways of searching and the contents of memory” in Autobiographical memory. ed. Rubin D. C. (Cambridge: Cambridge University Press), 50–67.

[ref113] Mandler G. (1980). Recognizing: the judgment of previous occurrence. Psychol. Rev. 87, 252–271. doi: 10.1037/0033-295X.87.3.252

[ref114] Markosian N. (2020). The dynamic theory of time and time travel to the past. Disputatio 12, 137–165. doi: 10.2478/disp-2020-0006

[ref115] Martin C. B. Deutscher M. (1966). Remembering. Philos. Rev. 75, 161–196. doi: 10.2307/2183082

[ref116] Matthen M. (2010). Is memory preservation? Philos. Stud. 148, 3–14. doi: 10.1007/s11098-010-9501-8

[ref117] Maylor E. A. Chater N. Brown G. D. (2001). Scale invariance in the retrieval of retrospective and prospective memories. Psychon. Bull. Rev. 8, 162–167. doi: 10.3758/BF03196153, PMID: 11340862

[ref118] Mazzoni G. Scoboria A. Harvey L. (2010). Nonbelieved memories. Psychol. Sci. 21, 1334–1340. doi: 10.1177/0956797610379865 20689053

[ref119] McCarroll C. J. (2018). Remembering from the outside: personal memory and the perspectival mind. Oxford: Oxford University Press.

[ref120] McCarroll C. J. Sutton J. (2017). “Memory and perspective” in The Routledge handbook of philosophy of memory. eds. Bernecker S. Michaelian K. (New York: Routledge), 113–126.

[ref121] McCormack T. (2001). “Attributing episodic memory to animals” in Time and memory: issues in philosophy and psychology. eds. Hoerl C. McCormack T. (New York: Oxford University Press), 285–313.

[ref122] McIsaac H. K. Eich E. (2004). Vantage point in traumatic memory. Psychol. Sci. 15, 248–253. doi: 10.1111/j.0956-7976.2004.00660.x, PMID: 15043642

[ref123] McNally R. J. (2012). Explaining “memories” of space alien abduction and past lives: an experimental psychopathology approach. J. Exp. Psychopathol. 3, 2–16. doi: 10.5127/jep.017811

[ref124] Meyersburg C. A. Carson S. H. Mathis M. B. McNally R. J. (2014). Creative histories: memories of past lives and measures of creativity. Psychol. Conscious. Theory Res. Pract. 1, 70–81. doi: 10.1037/css0000004

[ref125] Michaelian K. (2016). Mental time travel: episodic memory and our knowledge of the personal past. Cambridge: MIT Press.

[ref126] Miles A. N. Fischer-Mogensen L. Nielsen N. H. Hermansen S. Berntsen D. (2013). Turning back the hands of time: autobiographical memories in dementia cued by a museum setting. Conscious. Cogn. 22, 1074–1081. doi: 10.1016/j.concog.2013.07.008, PMID: 23948343

[ref127] Miller K. (2006). Travelling in time: how to wholly exist in two places at the same time. Can. J. Philos. 36, 309–334. doi: 10.1353/cjp.2006.0019

[ref128] Miller K. (2013). “Presentism, eternalism, and the growing block” in A companion to the philosophy of time. eds. Dyke H. Bardon A. (Malden: Wiley-Blackwell), 345–364.

[ref129] Miller K. (2017a). Is some backwards time travel inexplicable? Am. Philos. Q. 54, 131–140. doi: 10.2307/44982131

[ref130] Miller K. (2017b). Sorry, time travellers: you can’t change the past. Philos. Magaz. 77, 82–86. doi: 10.5840/tpm20177755

[ref131] Moscovitch M. (2007). “Memory: why the engram is ellusive” in Science of memory: concepts. eds. Roediger H. R. III Dudai Y. Fitzpatrick S. M. (New York: Oxford University Press), 17–22.

[ref132] Nadel L. (2007). “Consolidation: the demise of the fixed trace” in Science of memory: concepts. eds. Roediger H. L. III Dudai Y. Fitzpatrick S. M. (New York: Oxford University Press), 177–182.

[ref133] Nanay B. (2016). “Imagination and perception” in The Routledge handbook of philosophy of imagination. ed. Kind A. (New York: Routledge), 124–134.

[ref134] Neisser U. (1986). “Nested structure in autobiographical memory” in Autobiographical memory. ed. Rubin D. C. (Cambridge: Cambridge University Press), 71–81.

[ref135] Nikiforos C. D. Karakitsou C. (2020). The phenomenology of revisiting lived experience through photographic images: memory formation, narrative construction and self-empowerment. Qual. Rep. 25, 119–140. doi: 10.46743/2160-3715/2020.4763

[ref136] Nyberg L. Kim A. S. Habib R. Levine B. Tulving E. (2010). Consciousness of subjective time in the brain. Proc. Natl. Acad. Sci. U. S. A. 107, 22356–22359. doi: 10.1073/pnas.1016823108, PMID: 21135219 PMC3009795

[ref138] Palombo D. J. Sheldon S. Levine B. (2018). Individual differences in autobiographical memory. Trends Cogn. Sci. 22, 583–597. doi: 10.1016/j.tics.2018.04.007 29807853

[ref139] Patihis L. Cruz C. S. Herrera M. E. (2019). Changing current appraisals of mothers leads to changes in childhood memories of love toward mothers. Clin. Psychol. Sci. 7, 1125–1143. doi: 10.1177/2167702619842468

[ref140] Paulhan F. (1902). Sur la mémoire affective. Rev. Philos. France Let. 54, 545–569,

[ref141] Perrin D. Michaelian K. (2017). “Memory as mental time travel” in Routledge handbook of philosophy of memory. eds. Bernecker S. Michaelian K. (New York: Routledge), 228–239.

[ref142] Peters M. J. Horselenberg R. Jelicic M. Merckelbach H. (2007). The false fame illusion in people with memories about a previous life. Conscious. Cogn. 16, 162–169. doi: 10.1016/j.concog.2006.02.002, PMID: 16574433

[ref143] Piaget J. (1962) Play, Dreams and Imitation in Childhood. New York: W. W. Norton & Company, Inc.

[ref144] Piolino P. Desgranges B. Clarys D. Guillery-Girard B. Taconnat L. Isingrini M.. (2006). Autobiographical memory, autonoetic consciousness, and self-perspective in aging. Psychol. Aging 21, 510–525. doi: 10.1037/0882-7974.21.3.510, PMID: 16953713

[ref145] Piolino P. Desgranges B. Eustache F. (2009). Episodic autobiographical memories over the course of time: cognitive, neuropsychological and neuroimaging findings. Neuropsychologia 47, 2314–2329. doi: 10.1016/j.neuropsychologia.2009.01.020, PMID: 19524095

[ref146] Proust M. (1928). Swann’s way. New York, NY: Modern Library.

[ref147] Radley A. Taylor D. (2003). Remembering one’s stay in hospital: a study in photography, recovery and forgetting. Health 7, 129–159. doi: 10.1177/1363459303007002872

[ref148] Rawls C. Miller K. (2000). “Time will tell: an interview with Kristie Miller” in Blog of the American philosophical association (APA). Available at: https://blog.apaonline.org/2020/09/04/time-will-tell-an-interview-with-kristie-miller/

[ref149] Ribot T. (1907). La mémoire affective: nouvelles remarques. Rev. Philos. France l’Etranger 64, 588–613,

[ref150] Rice H. J. Rubin D. C. (2009). I can see it both ways: first- and third-person visual perspectives at retrieval. Conscious. Cogn. 18, 877–890. doi: 10.1016/j.concog.2009.07.004, PMID: 19692271 PMC2784183

[ref151] Robins S. K. (2017). “Memory traces” in The Routledge handbook of philosophy of memory. eds. Bernecker S. Michaelian K. (New York: Routledge), 76–87.

[ref152] Robins S. K. (2018). Memory and optogenetic intervention: separating the engram from the ecphory. Philos. Sci. 85, 1078–1089. doi: 10.1086/699692

[ref153] Robins S. K. (2019). Confabulation and constructive memory. Synthese 196, 2135–2151. doi: 10.1007/s11229-017-1315-1

[ref154] Robins S. K. (2020a). Stable engrams and neural dynamics. Philos. Sci. 87, 1130–1139. doi: 10.1086/710624

[ref155] Robins S. K. (2020b). Mnemonic confabulation. Topoi 39, 121–132. doi: 10.1007/s11245-018-9613-x

[ref156] Robinson J. A. Swanson K. L. (1993). Field and observer modes of remembering. Memory 1, 169–184. doi: 10.1080/09658219308258230, PMID: 7584265

[ref157] Roediger H. L. (1980). Memory metaphors in cognitive psychology. Mem. Cogn. 8, 231–246. doi: 10.3758/BF03197611 7392950

[ref158] Roese N. J. Epstude K. (2017). “The functional theory of counterfactual thinking: new evidence, new challenges, new insights” in Advances in experimental social psychology. ed. Olson J., Vol. 56 (Academic Press), 1–79.

[ref159] Rosen M. G. Barkasi M. (2021). What makes a mental state feel like a memory: feelings of pastness and presence. Estud. Filos. 64, 95–122. doi: 10.17533/udea.ef.n64a05

[ref160] Rubin D. C. Berntsen D. (2009). The frequency of voluntary and involuntary autobiographical memories across the life span. Mem. Cogn. 37, 679–688. doi: 10.3758/37.5.679, PMID: 19487759 PMC3044938

[ref161] Rubin D. C. Boals A. Berntsen D. (2008). Memory in posttraumatic stress disorder: properties of voluntary and involuntary, traumatic and nontraumatic autobiographical memories in people with and without posttraumatic stress disorder symptoms. J. Exp. Psychol. Gen. 137, 591–614. doi: 10.1037/a0013165, PMID: 18999355 PMC2597428

[ref162] Rubin D. C. Deffler S. A. Umanath S. (2019). Scenes enable a sense of reliving: implications for autobiographical memory. Cognition 183, 44–56. doi: 10.1016/j.cognition.2018.10.024, PMID: 30412854 PMC6322930

[ref163] Rubin D. C. Schrauf R. W. Greenberg D. L. (2003). Belief and recollection of autobiographical memories. Mem. Cogn. 31, 887–901. doi: 10.3758/BF03196443 14651297

[ref164] Rubin D. C. Umanath S. (2015). Event memory: a theory of memory for laboratory, autobiographical, and fictional events. Psychol. Rev. 122, 1–23. doi: 10.1037/a0037907, PMID: 25330330 PMC4295926

[ref165] Russell B. (1921). The analysis of mind. London: George Allen & Unwin.

[ref166] Russell J. A. (2009). Emotion, core affect, and psychological construction. Cognit. Emot. 23, 1259–1283. doi: 10.1080/02699930902809375

[ref167] Russell J. A. Barrett L. F. (1999). Core affect, prototypical emotional episodes, and other things called emotion: dissecting the elephant. J. Pers. Soc. Psychol. 76, 805–819. doi: 10.1037/0022-3514.76.5.805, PMID: 10353204

[ref169] Schechtman M. (2001). Empathic access: the missing ingredient in personal identity. Philos. Explor. 4, 95–111. doi: 10.1080/10002001058538710

[ref170] Schooler J. W. Smallwood J. Christoff K. Handy T. C. Reichle E. D. Sayette M. A. (2011). Meta-awareness, perceptual decoupling and the wandering mind. Trends Cogn. Sci. 15, 319–326. doi: 10.1016/j.tics.2011.05.006, PMID: 21684189

[ref171] Semon R. W. (1921). The Mneme. London: Allen & Unwin.

[ref172] Sharrock W. Leudar I. (2002). Indeterminacy in the past? Hist. Hum. Sci. 15, 95–115. doi: 10.1177/0952695102015003169

[ref173] Smallwood J. Schooler J. W. (2015). The science of mind wandering: empirically navigating the stream of consciousness. Annu. Rev. Psychol. 66, 487–518. doi: 10.1146/annurev-psych-010814-015331, PMID: 25293689

[ref174] Smith S. T. (2013). “Effects of environmental context on human memory” in The Sage handbook of applied memory. eds. Perfect T. J. Lindsay D. S. (Sage Publications), 162–182.

[ref175] Smith-Spark J. H. Bartimus J. Wilcock R. (2017). Mental time travel ability and the mental reinstatement of context for crime witnesses. Conscious. Cogn. 48, 1–10. doi: 10.1016/j.concog.2016.10.002, PMID: 27788399

[ref176] Springora V. (2020). Le consentement. Paris: Le livre de Poche.

[ref177] Suddendorf T. Corballis M. C. (1997). Mental time travel and the evolution of the human mind. Genet. Soc. Gen. Psychol. Monogr. 123, 133–167, 9204544

[ref178] Suddendorf T. Corballis M. C. (2007). The evolution of foresight: what is mental time travel, and is it unique to humans? Behav. Brain Sci. 30, 299–313. doi: 10.1017/S0140525X07001975, PMID: 17963565

[ref179] Sutton J. (2010). Observer perspective and acentred memory: some puzzles about point of view in personal memory. Philos. Stud. 148, 27–37. doi: 10.1017/9781139424615

[ref180] Sutton J. (2014). Memory perspectives. Mem. Stud. 7, 141–145. doi: 10.1177/1750698013518131

[ref181] Sutton J. O’Brien G. (2022). “Distributed traces and the causal theory of constructive memory” in Current controversies in philosophy of memory. eds. Sant’Anna A. McCarroll C. J. Michaelian K. (Abingdon: Routledge).

[ref183] Talarico J. M. LaBar K. S. Rubin D. C. (2004). Emotional intensity predicts autobiographical memory experience. Mem. Cogn. 32, 1118–1132. doi: 10.3758/BF03196886, PMID: 15813494

[ref184] Tekcan A. İ. Yılmaz E. Kaya Kızılöz B. Karadöller D. Z. Mutafoğlu M. Aktan Erciyes A. (2015). Retrieval and phenomenology of autobiographical memories in blind individuals. Memory 23, 329–339. doi: 10.1080/09658211.2014.886702, PMID: 24528249

[ref185] Trakas M. (2015). Personal memories. PhD thesis, Macquarie University and Ecole des hautes études en sciences sociales. Available at: http://hdl.handle.net/1959.14/1069913

[ref186] Trakas M. (2019a). How to distinguish memory representations? A historical and critical journey. Voluntas 10, 53–86. doi: 10.5902/2179378639849

[ref187] Trakas M. (2019b). On epistemic responsibility while remembering the past: the case of individual and historical memories. Ethics Forum 14, 240–273. doi: 10.7202/1071139ar

[ref188] Trakas M. (2021a). Kinetic memories. An embodied form of remembering the personal past. J Mind Beha. 42, 139–174,

[ref189] Trakas M. (2021b). Dimensiones de análisis de los recuerdos personales como recuerdos afectivos. Rev. Psicol. 20, 256–284. doi: 10.24215/2422572Xe126

[ref190] Trakas M. (2021c). The sense of mineness in personal memory: problems for the endorsement model. Estud. Filos. 64, 155–172. doi: 10.17533/udea.ef.n64a08

[ref191] Trakas M. (2022). El viaje mental en el tiempo en la filosofía y la ciencia cognitiva de la memoria. Rev. Human. Valpar. 20, 141–163. doi: 10.22370/rhv2022iss20pp141-163

[ref192] Tulving E. (1983). Elements of episodic memory. Clarendon Press.

[ref193] Tulving E. (1985). Memory and consciousness. Can. Psychol. 26, 1–12. doi: 10.1037/h0080017

[ref194] Tulving E. (2002a). Episodic memory: from mind to brain. Annu. Rev. Psychol. 53, 1–25. doi: 10.1146/annurev.psych.53.100901.135114 11752477

[ref195] Tulving E. (2002b). “Chronesthesia: conscious awareness of subjective time” in Principles of frontal lobe function. eds. Stuss D. T. Knight R. T. (New York: Oxford University Press), 311–325.

[ref196] Tulving E. (2005). “Episodic memory and autonoesis: uniquely human?” in The missing link in cognition: origins of self-reflective consciousness. eds. Terrace H. S. Metcalfe J. (New York: Oxford University Press), 3–56.

[ref197] Tulving E. Thomson D. M. (1973). Encoding specificity and retrieval processes in episodic memory. Psychol. Rev. 80, 352–373. doi: 10.1037/h0020071

[ref198] Urban E. J. Charles S. T. Levine L. J. Almeida D. M. (2018). Depression history and memory bias for specific daily emotions. PLoS One 13:e0203574. doi: 10.1371/2Fjournal.pone.0203574, PMID: 30192853 PMC6128594

[ref199] Van Inwagen P. (2010). “Changing the past” in Oxford studies in metaphysics. ed. Zimmerman D., Vol. 5 (Oxford: Oxford University Press), 3–40.

[ref200] Weber L. (1914). Sur la mémoire affective. Rev. Metaphys. Morale 22, 794–813,

[ref201] Wells H. G. (1895). The time machine. London: William Heinemann Ltd.

[ref202] Wheeler M. A. Stuss D. T. Tulving E. (1997). Toward a theory of episodic memory: the frontallobes and autonoetic consciousness. Psychol. Bull. 121, 331–354. doi: 10.1037/0033-2909.121.3.331, PMID: 9136640

[ref203] Willander J. Larsson M. (2007). Olfaction and emotion: the case of autobiographical memory. Mem. Cogn. 35, 1659–1663. doi: 10.3758/BF03193499, PMID: 18062543

[ref204] Wollheim R. (1984). The thread of life. Glasgow: Harvard University Press.

[ref205] Woozley A. D. (1949). Theory of knowledge. London: Hutchinson of London.

[ref206] Zaman A. Russell C. (2022). Does autonoetic consciousness in episodic memory rely on recall from a first-person perspective? J. Cogn. Psychol. 34, 9–23. doi: 10.1080/20445911.2021.1922419

[ref207] Zeman A. Z. Della Sala S. Torrens L. A. Gountouna V. E. McGonigle D. J. Logie R. H. (2010). Loss of imagery phenomenology with intact visuo-spatial task performance: a case of ‘blind imagination’. Neuropsychologia 48, 145–155. doi: 10.1016/j.neuropsychologia.2009.08.024, PMID: 19733188

[ref208] Zeman A. Dewar M. Della Sala S. (2015). Lives without imagery–congenital aphantasia. Cortex 73, 378–380. doi: 10.1016/j.cortex.2015.05.019, PMID: 26115582

[ref209] Zeman A. Milton F. Della S. S. Dewar M. Frayling T. Gaddum J.. (2020). Phantasia—the psychological significance of lifelong visual imagery vividness extremes. Cortex 130, 426–440. doi: 10.1093/texcom/tgab035, PMID: 32446532

